# Supplementary material for: Proteomic Analysis of PTEN-Deficient Cells Reveals Src-Mediated Upregulation of EphA2 and Therapeutic Potential of Dual Inhibition
Source: Mol Cell Proteomics. 2025 Oct 21;24(12):101316. doi: 10.1016/j.mcpro.2025.101316 (PMC12686823; doi:10.1016/j.mcpro.2025.101316)
Supplement: Supplementary Images [file mmc10.pdf]

Fig. 1A

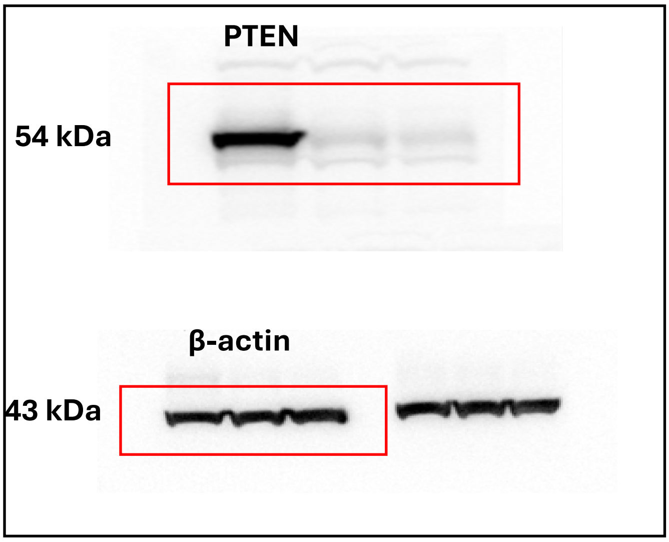
PTEN
54 kDa
β-actin
43 kDa

Fig. 1C

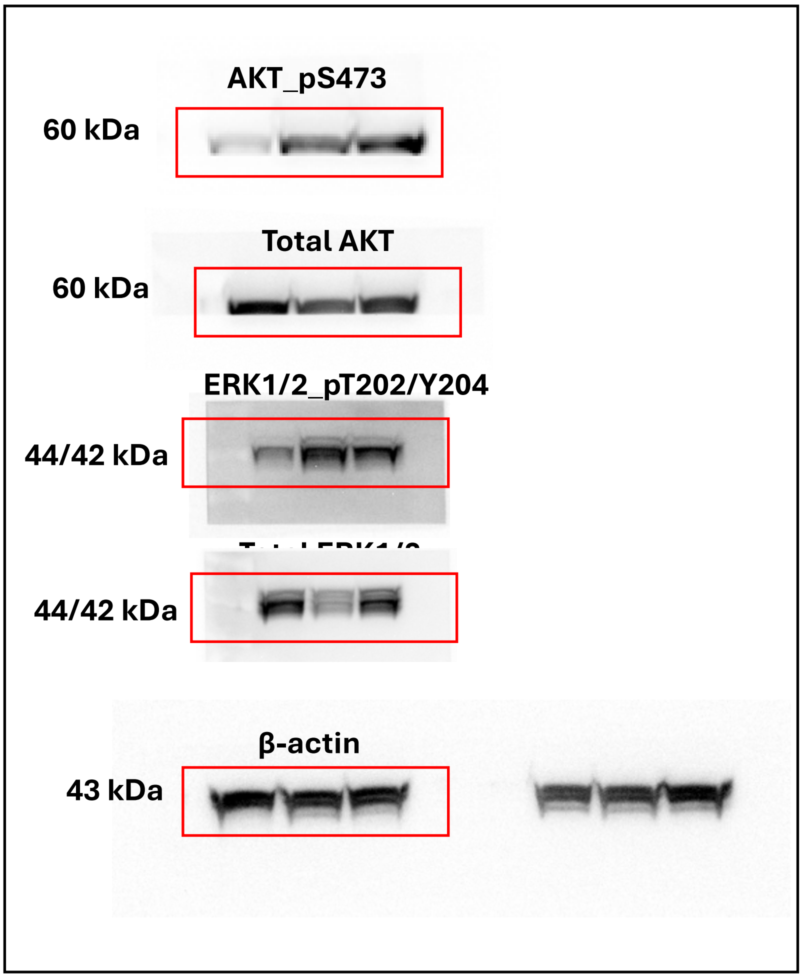
AKT_pS473
60 kDa
Total AKT
60 kDa
ERK1/2_pT202/Y204
44/42 kDa
44/42 kDa
β-actin
43 kDa

Fig. 3J

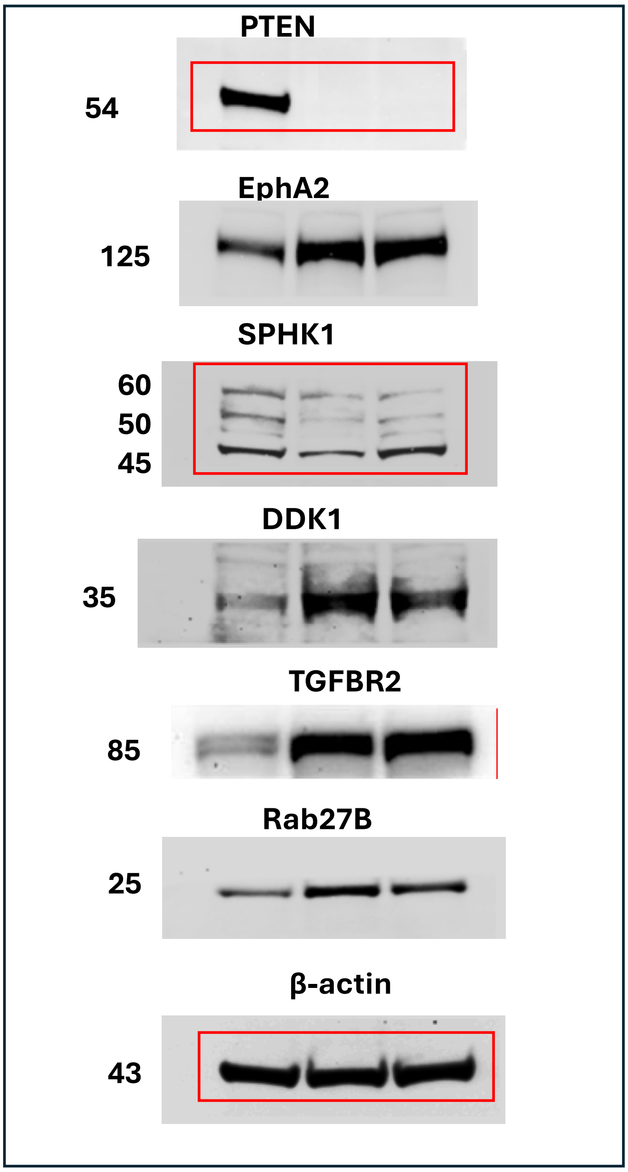
PTEN
54
EphA2
125
SPHK1
60
50
45
DDK1
35
TGFBR2
85
Rab27B
25
β-actin
43

Fig. 5A

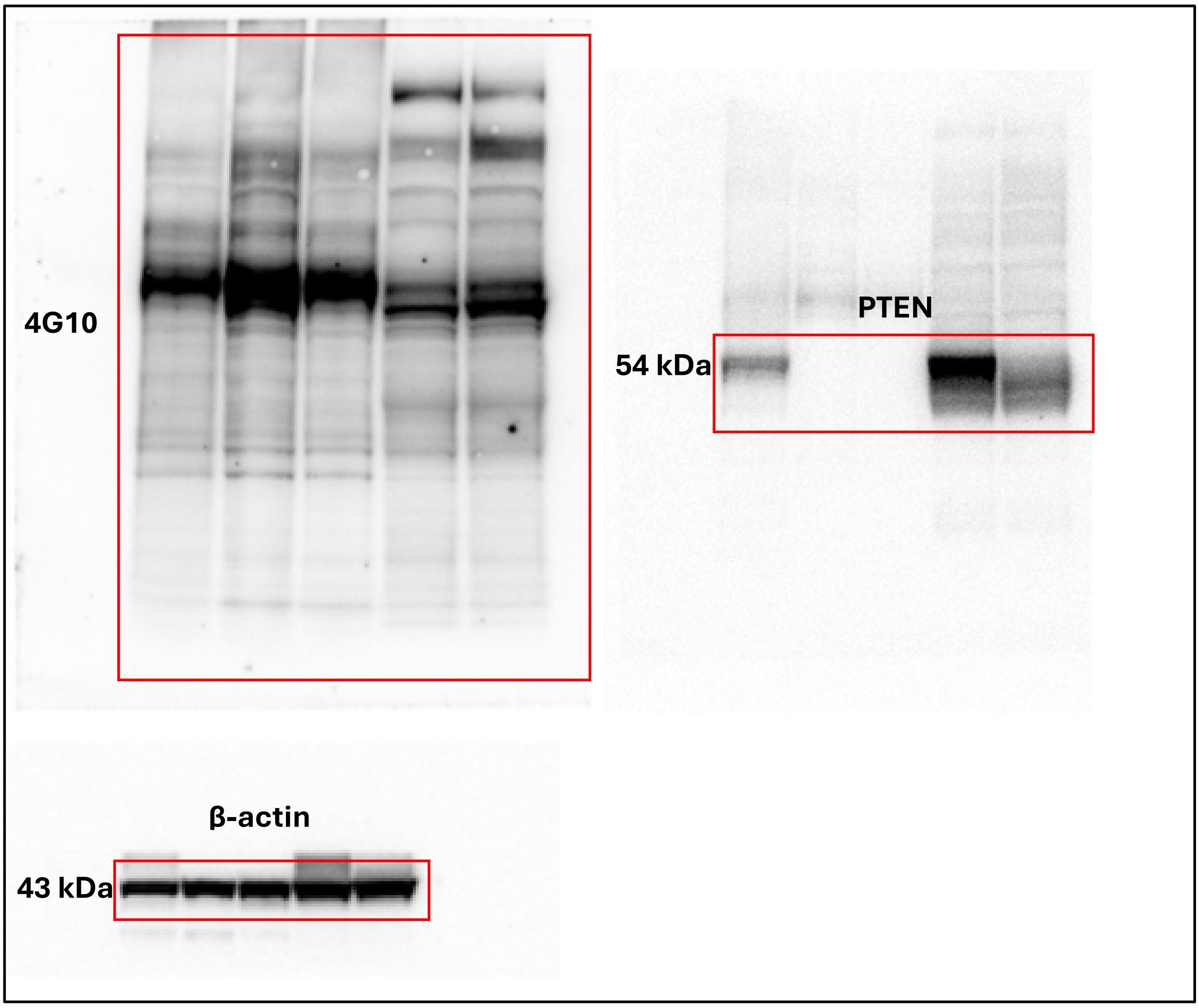
4G10
PTEN
54 kDa
β-actin
43 kDa

Fig. 6C

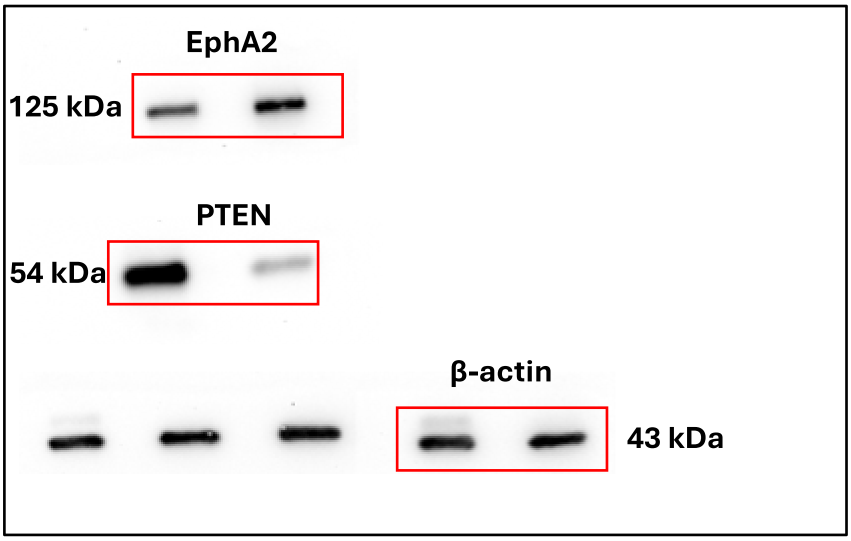
EphA2
125 kDa
PTEN
54 kDa
β-actin
43 kDa

Fig. 6D

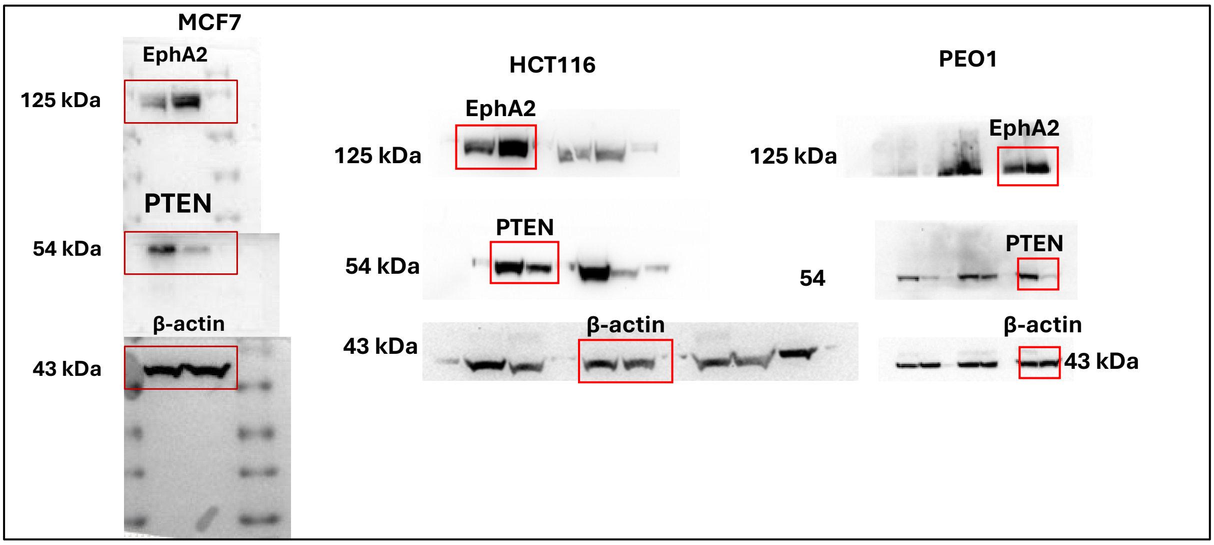
MCF7
EphA2
125 kDa
PTEN
54 kDa
β-actin
43 kDa
HCT116
EphA2
125 kDa
PTEN
54 kDa
β-actin
43 kDa
PEO1
EphA2
125 kDa
PTEN
54
β-actin
43 kDa

Fig. 6E

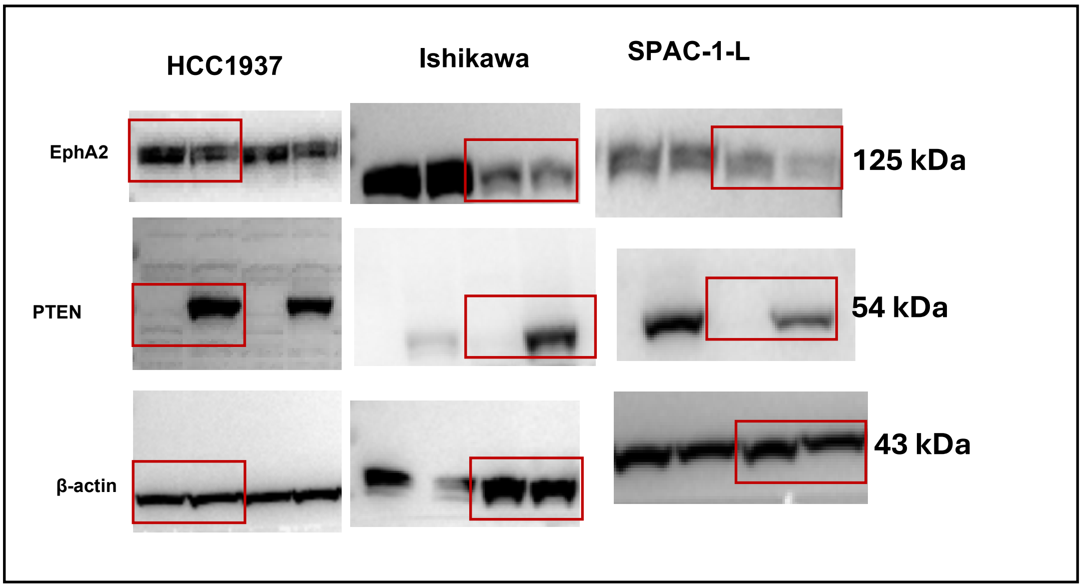
HCC1937
Ishikawa
SPAC-1-L
EphA2
125 kDa
PTEN
54 kDa
β-actin
43 kDa

Fig. 6G

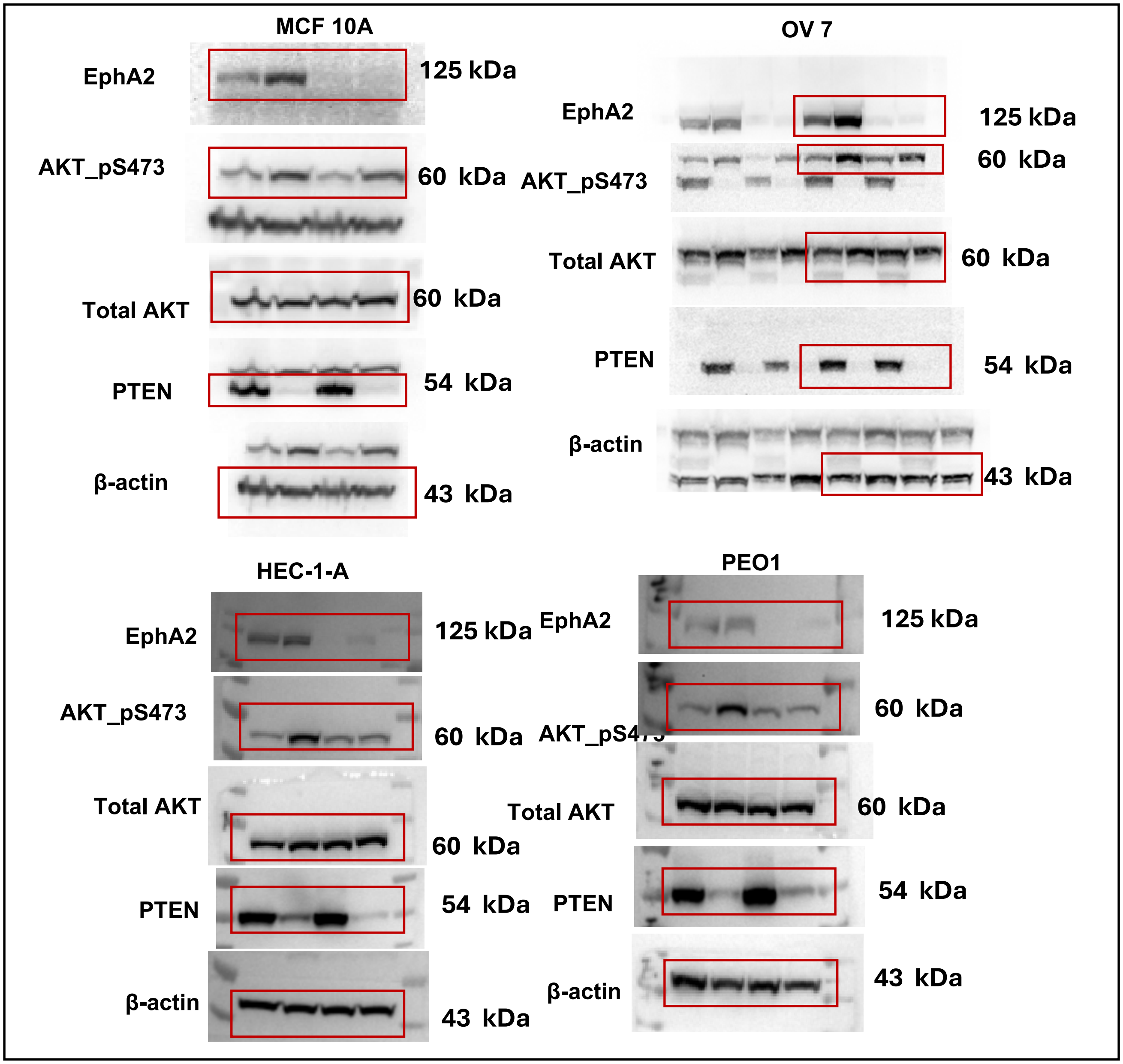
MCF 10A
EphA2
125 kDa
AKT_pS473
60 kDa
Total AKT
60 kDa
PTEN
54 kDa
β-actin
43 kDa
OV 7
EphA2
125 kDa
AKT_pS473
60 kDa
Total AKT
60 kDa
PTEN
54 kDa
β-actin
43 kDa
HEC-1-A
EphA2
125 kDa
AKT_pS473
60 kDa
Total AKT
60 kDa
PTEN
54 kDa
β-actin
43 kDa
PEO1
EphA2
125 kDa
60 kDa
Total AKT
60 kDa
PTEN
54 kDa
β-actin
43 kDa

Fig. 6J

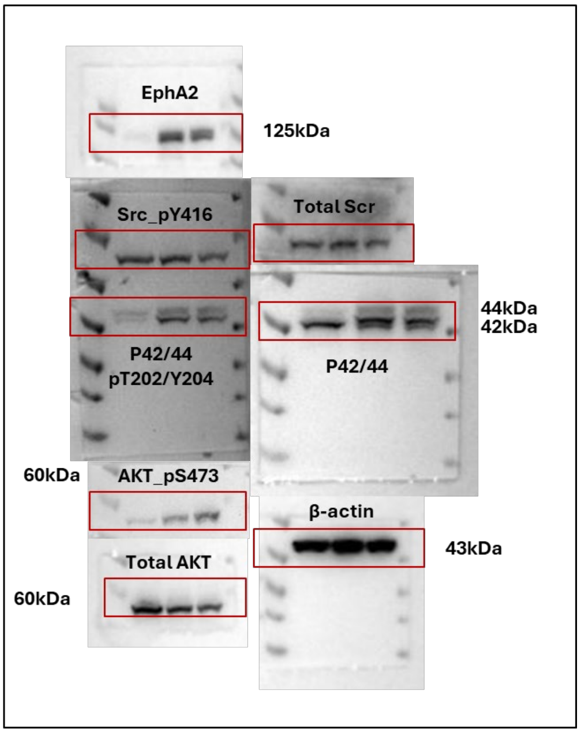

EphA2
125kDa
Src_pY416
Total Scr
44kDa
42kDa
P42/44
pT202/Y204
P42/44
60kDa
AKT_pS473
β-actin
43kDa
Total AKT
60kDa

Fig. 6K

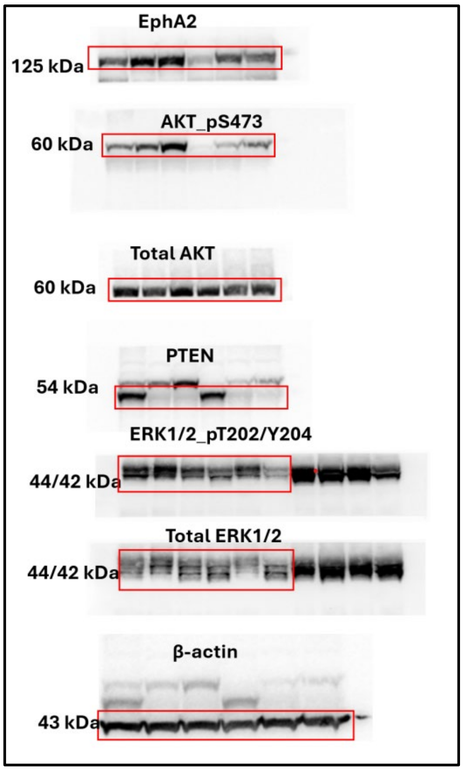

EphA2
125 kDa
AKT_pS473
60 kDa
Total AKT
60 kDa
PTEN
54 kDa
ERK1/2_pT202/Y204
44/42 kDa
Total ERK1/2
44/42 kDa
β-actin
43 kDa

Fig. 6L

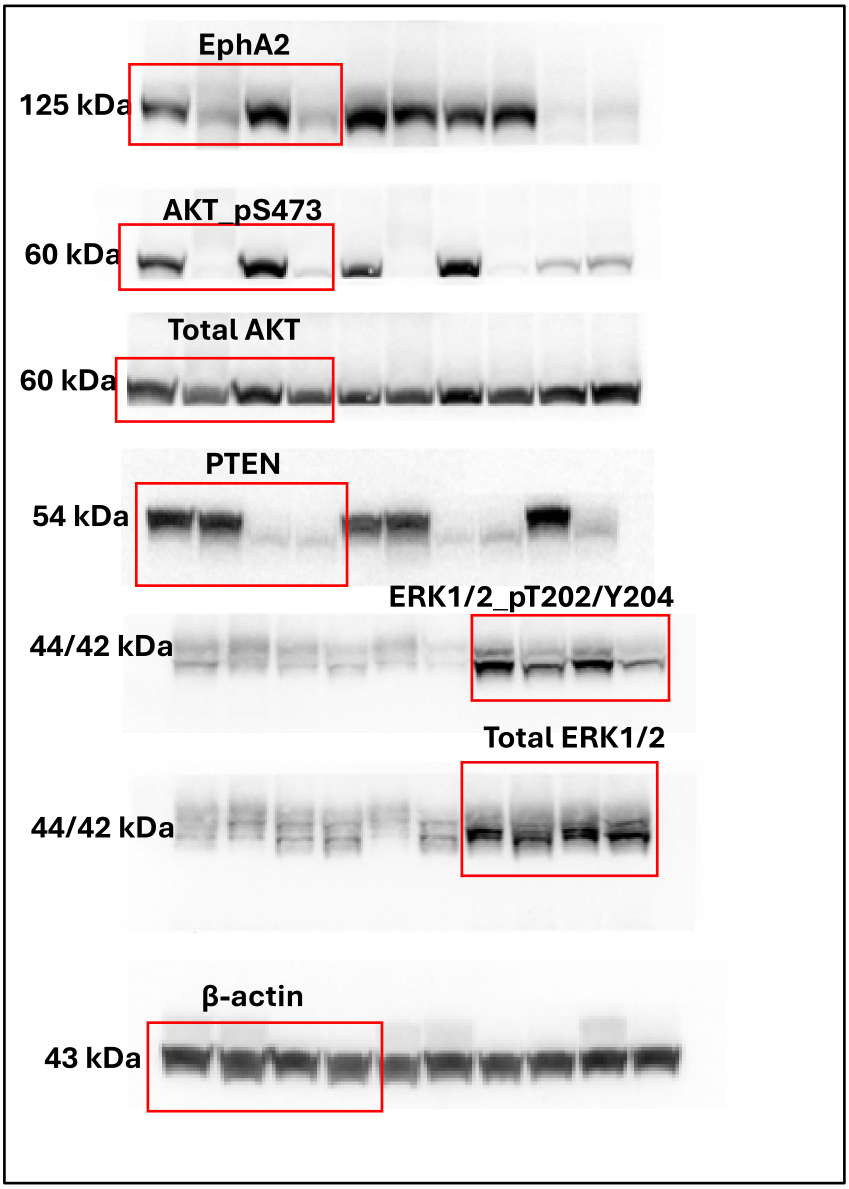
EphA2
125 kDa
AKT_pS473
60 kDa
Total AKT
60 kDa
PTEN
54 kDa
ERK1/2_pT202/Y204
44/42 kDa
Total ERK1/2
44/42 kDa
β-actin
43 kDa

Fig. 6M

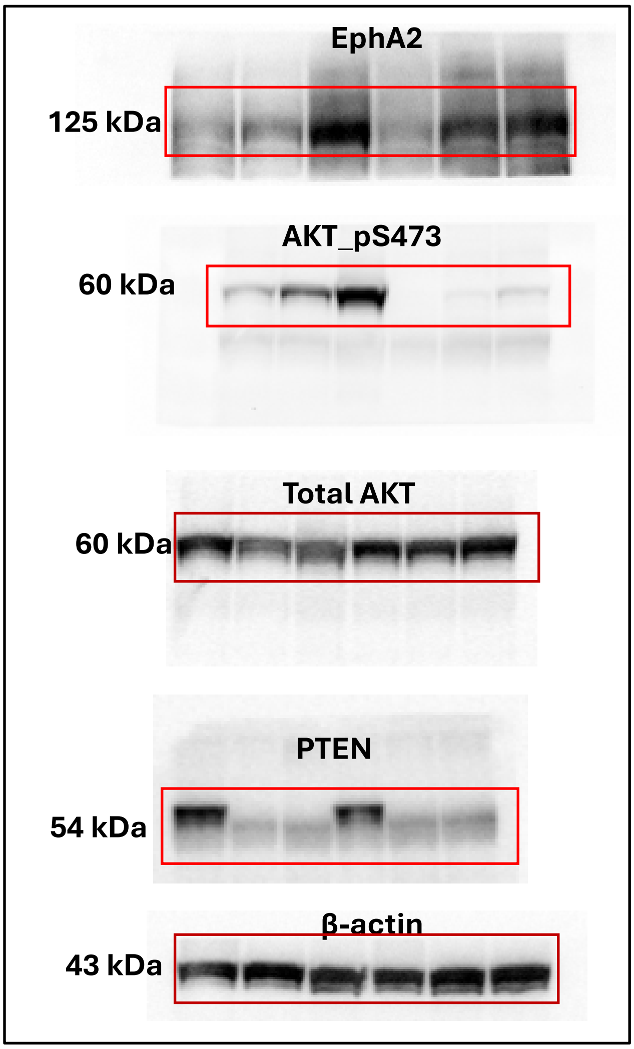

EphA2
125 kDa
AKT_pS473
60 kDa
Total AKT
60 kDa
PTEN
54 kDa
β-actin
43 kDa

Fig. 6N

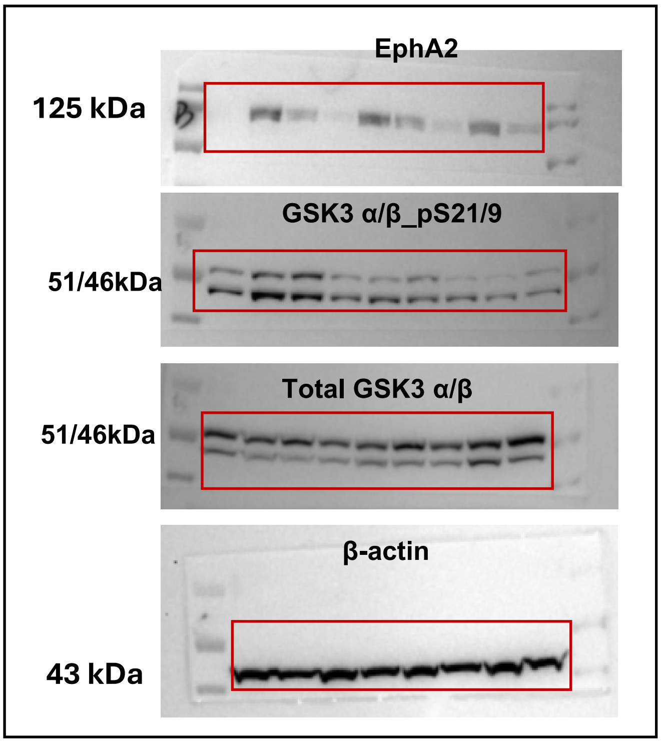

EphA2
125 kDa
GSK3 α/β_pS21/9
51/46kDa
Total GSK3 α/β
51/46kDa
β-actin
43 kDa

Fig. 6O

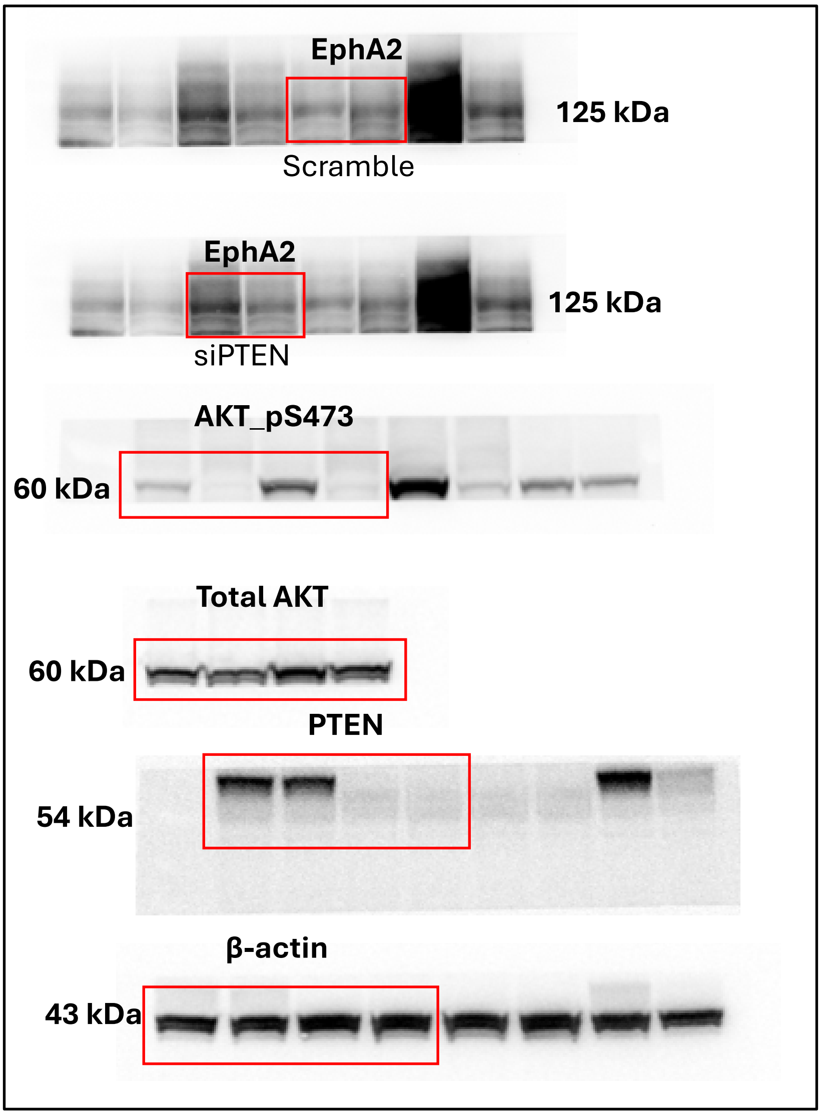
EphA2
125 kDa
Scramble
EphA2
125 kDa
siPTEN
AKT_pS473
60 kDa
Total AKT
60 kDa
PTEN
54 kDa
β-actin
43 kDa

Fig. 7B

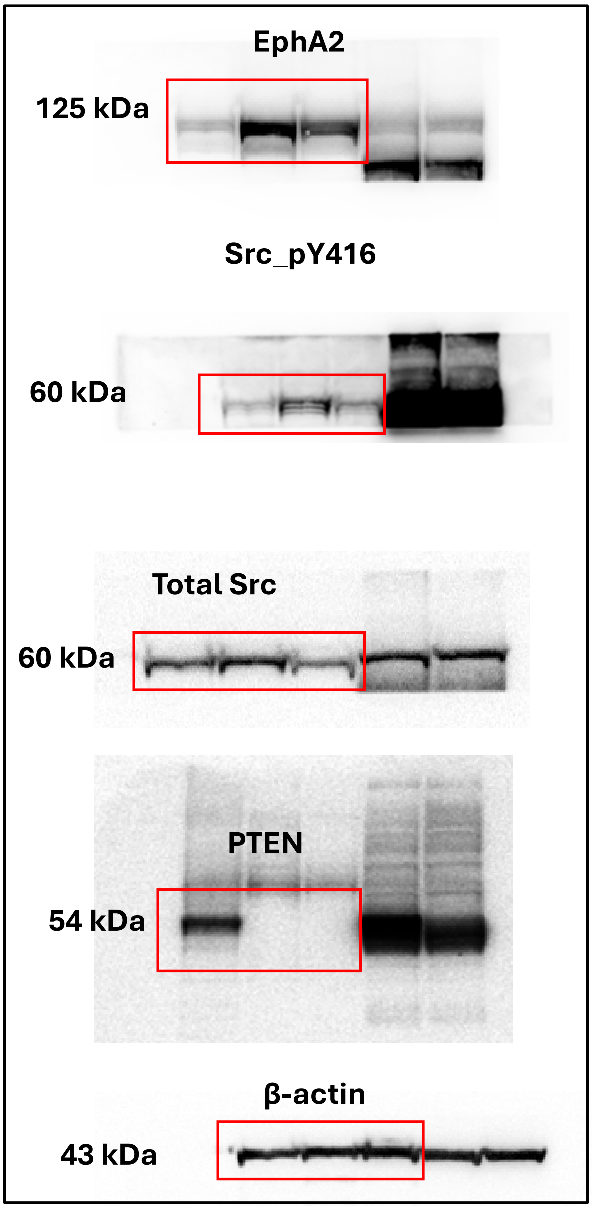

Fig. 7C

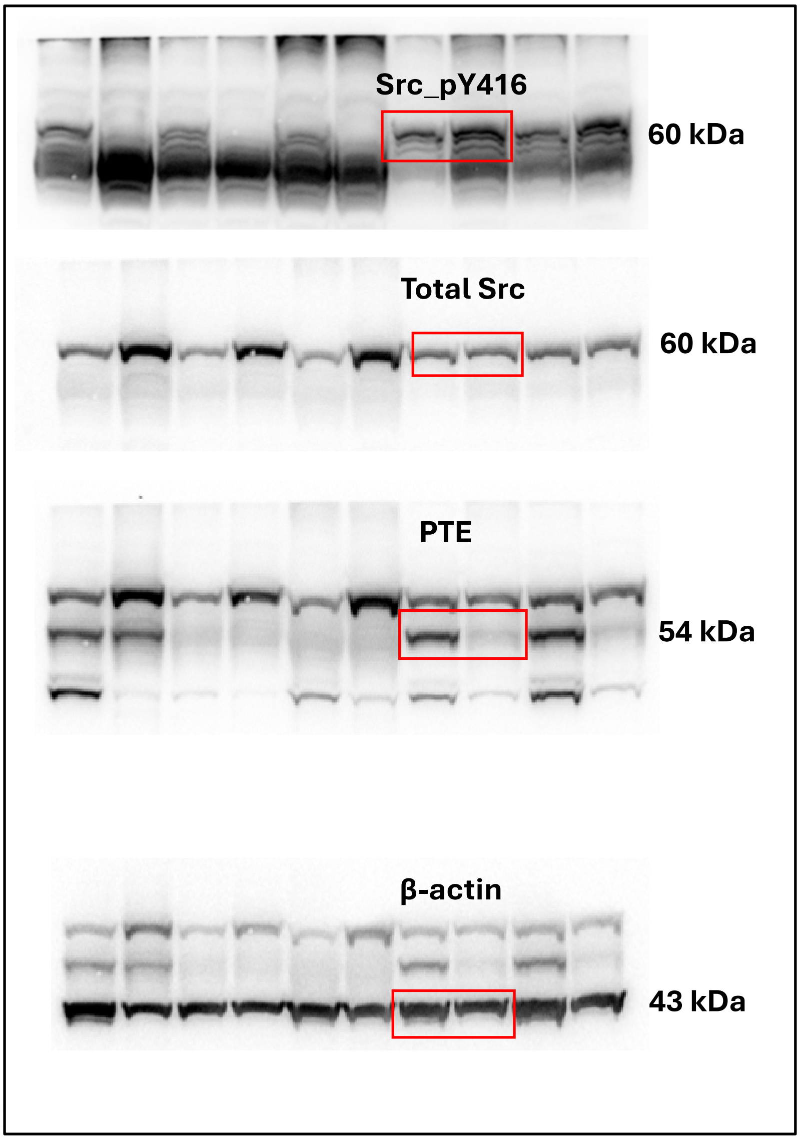

Fig. 7D

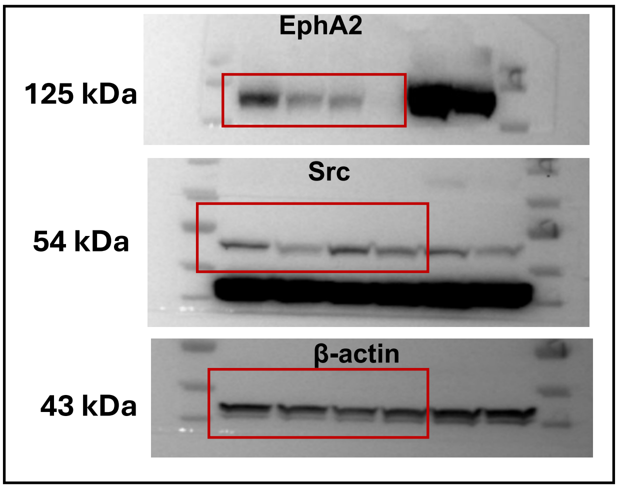

Fig. 7E

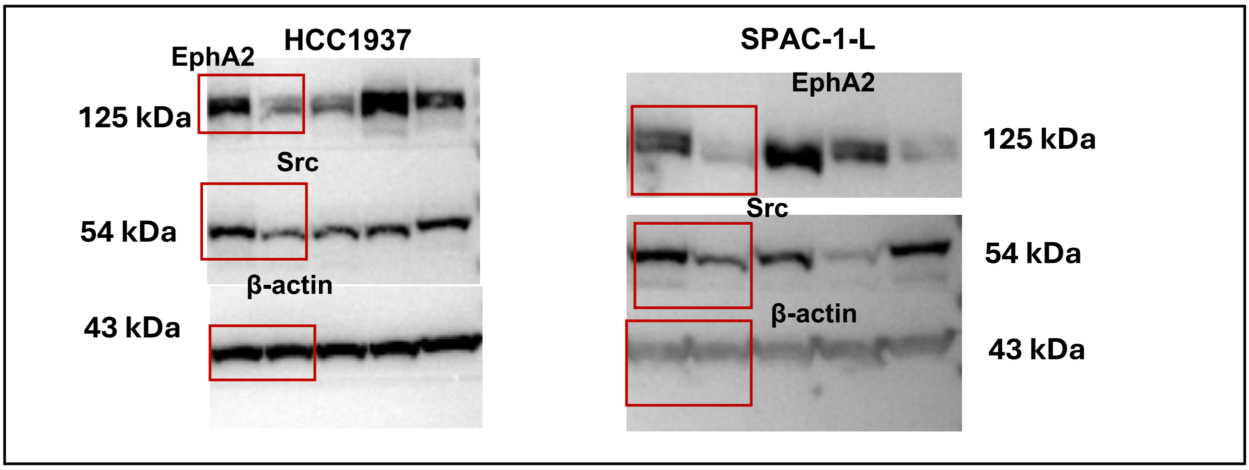
HCC1937
EphA2
125 kDa
Src
54 kDa
β-actin
43 kDa
SPAC-1-L
EphA2
125 kDa
Src
54 kDa
β-actin
43 kDa

Fig. 7F

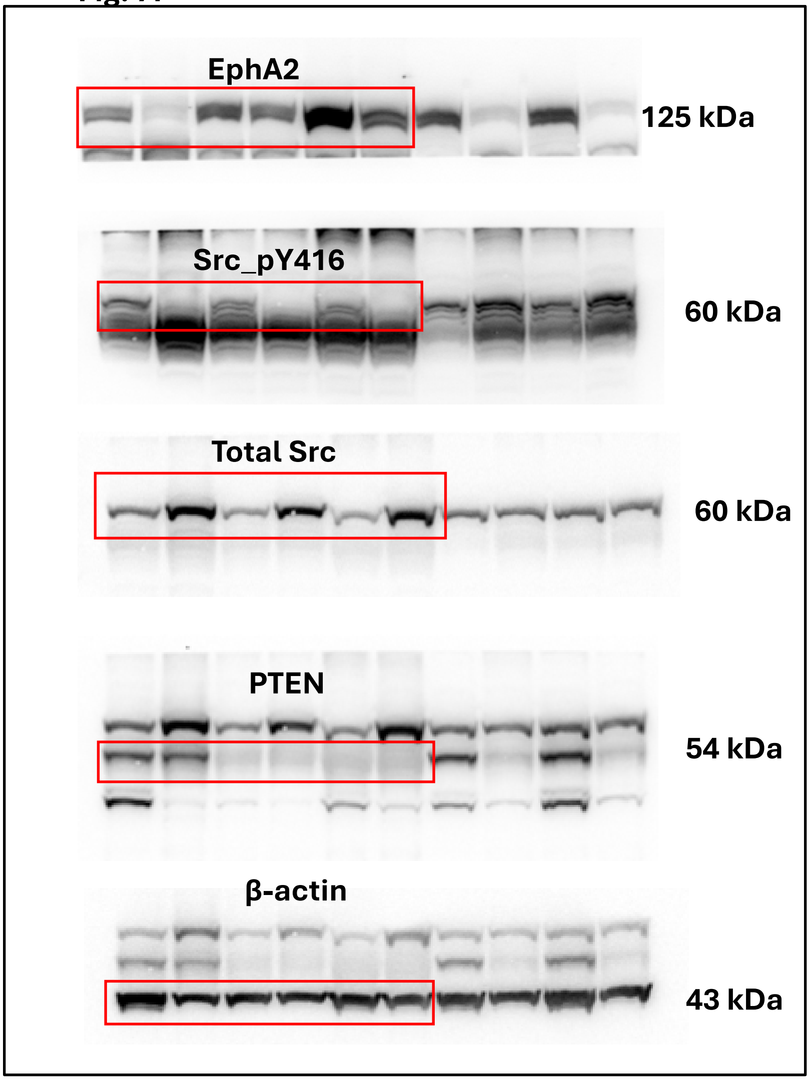
EphA2
125 kDa
Src_pY416
60 kDa
Total Src
60 kDa
PTEN
54 kDa
β-actin
43 kDa

Fig. 7G

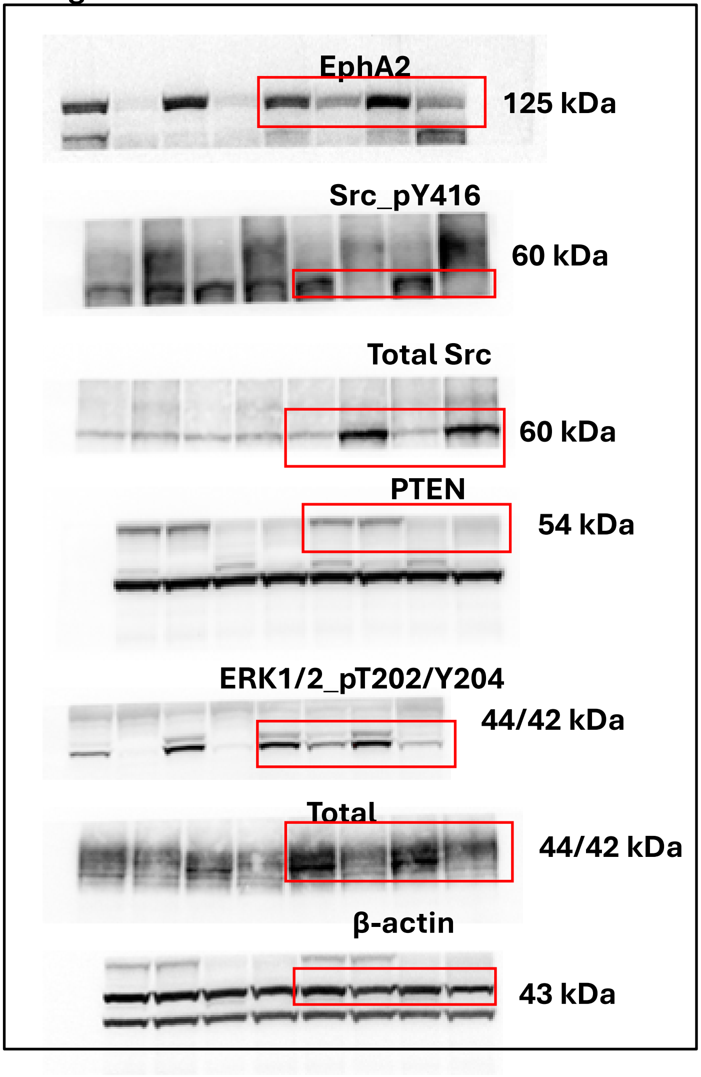
EphA2
125 kDa
Src_pY416
60 kDa
Total Src
60 kDa
PTEN
54 kDa
ERK1/2_pT202/Y204
44/42 kDa
Total
44/42 kDa
β-actin
43 kDa

Fig. 7H

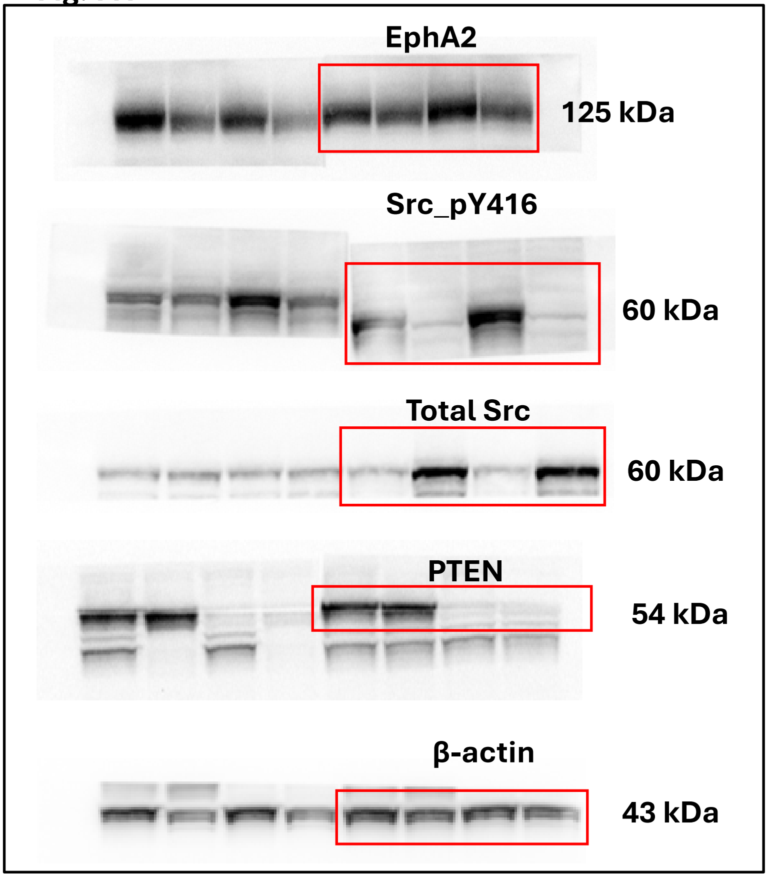
EphA2
125 kDa
Src_pY416
60 kDa
Total Src
60 kDa
PTEN
54 kDa
β-actin
43 kDa

Fig. 7I

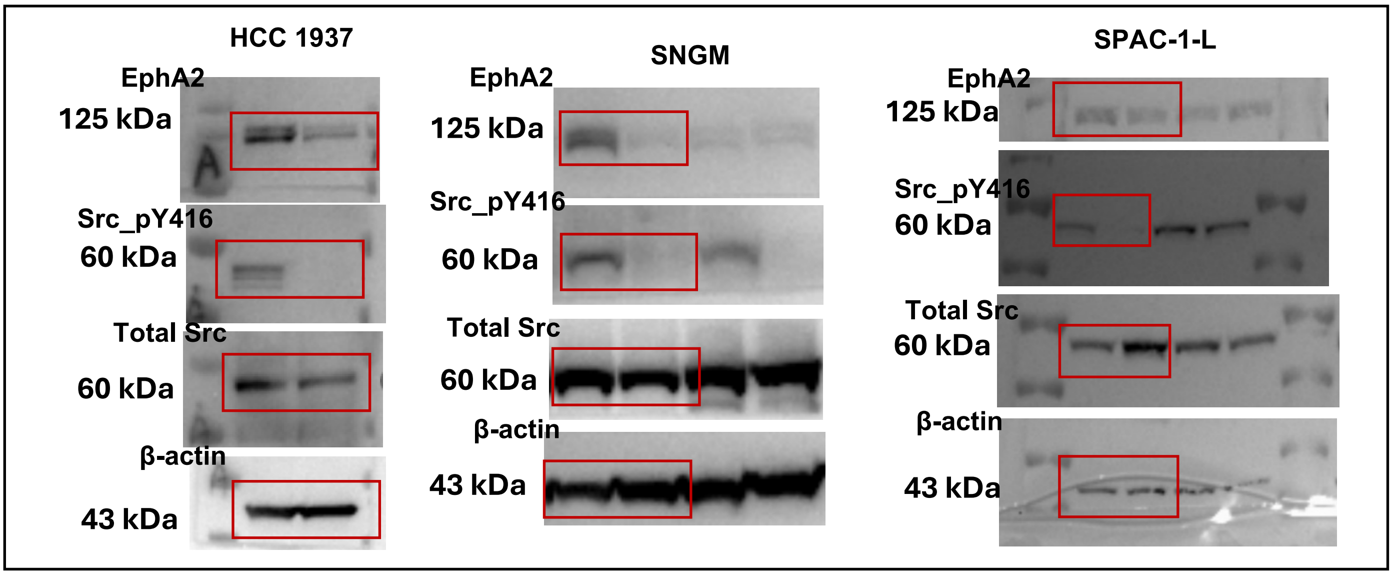
HCC 1937
EphA2
125 kDa
Src_pY416
60 kDa
Total Src
60 kDa
β-actin
43 kDa
SNGM
EphA2
125 kDa
Src_pY416
60 kDa
Total Src
60 kDa
β-actin
43 kDa
SPAC-1-L
EphA2
125 kDa
Src_pY416
60 kDa
Total Src
60 kDa
β-actin
43 kDa

Fig. 7J

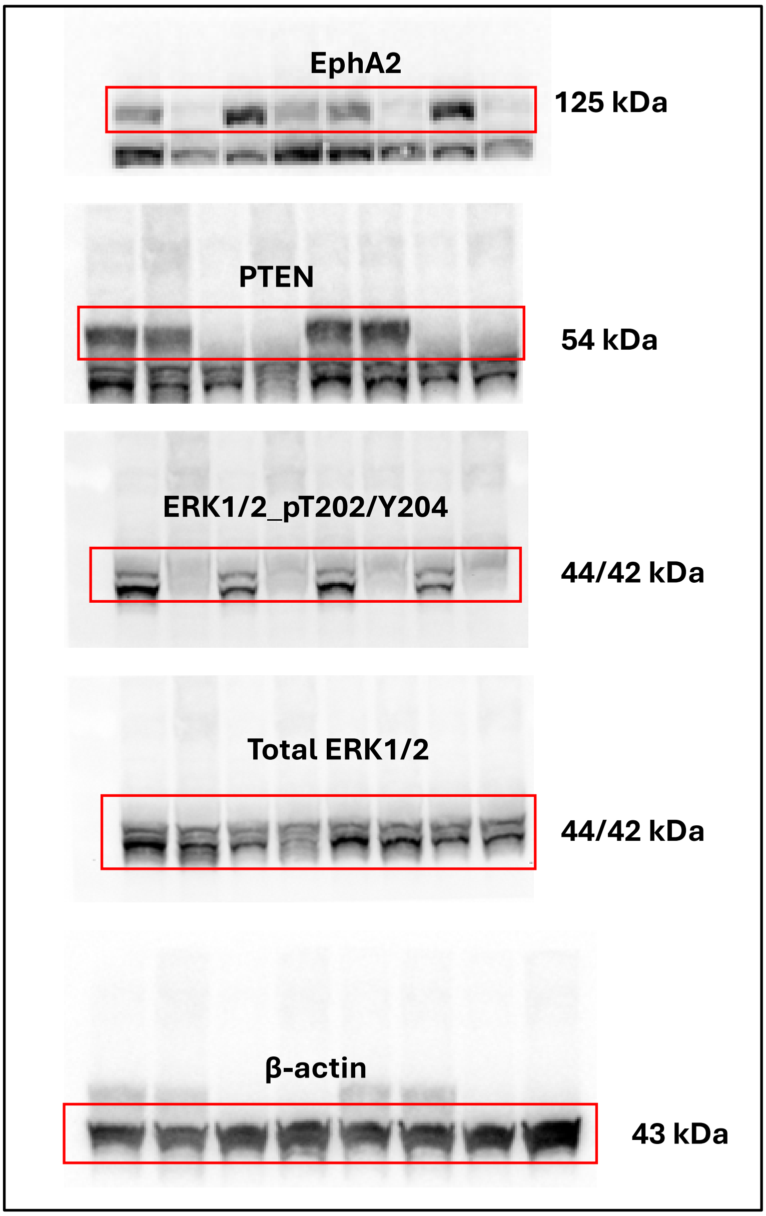

EphA2
125 kDa
PTEN
54 kDa
ERK1/2_pT202/Y204
44/42 kDa
Total ERK1/2
44/42 kDa
β-actin
43 kDa

Fig. 7K

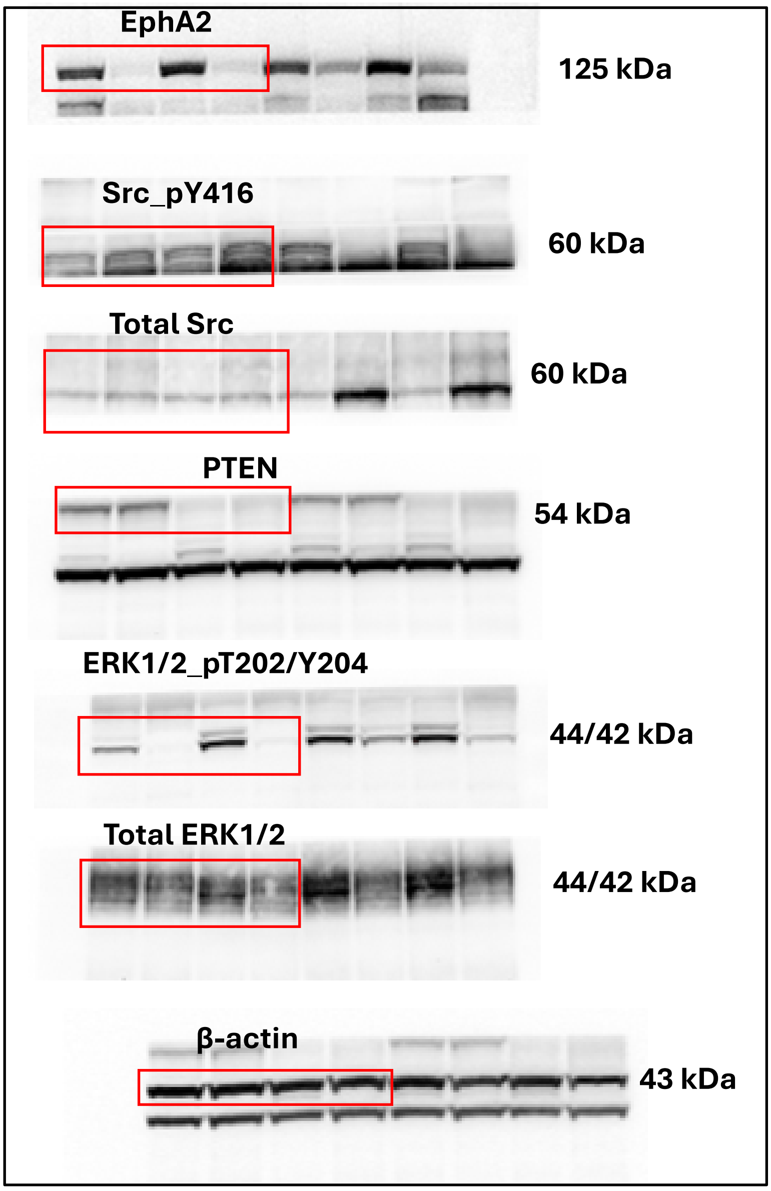
EphA2
125 kDa
Src_pY416
60 kDa
Total Src
60 kDa
PTEN
54 kDa
ERK1/2_pT202/Y204
44/42 kDa
Total ERK1/2
44/42 kDa
β-actin
43 kDa

Fig. 7L

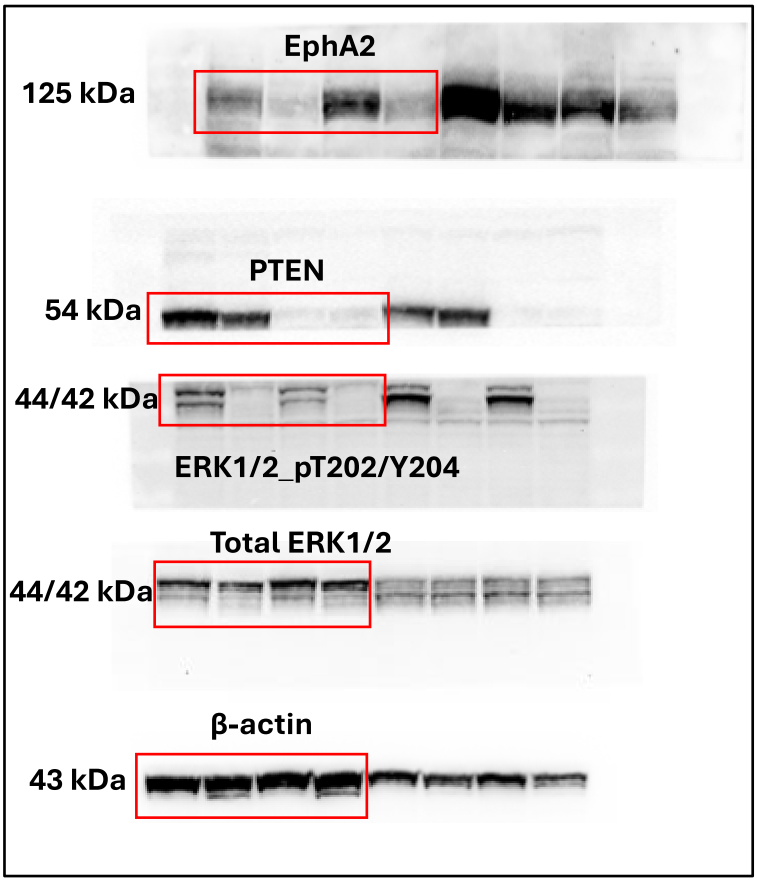
EphA2
125 kDa
PTEN
54 kDa
44/42 kDa
ERK1/2_pT202/Y204
Total ERK1/2
44/42 kDa
β-actin
43 kDa

Fig 8D

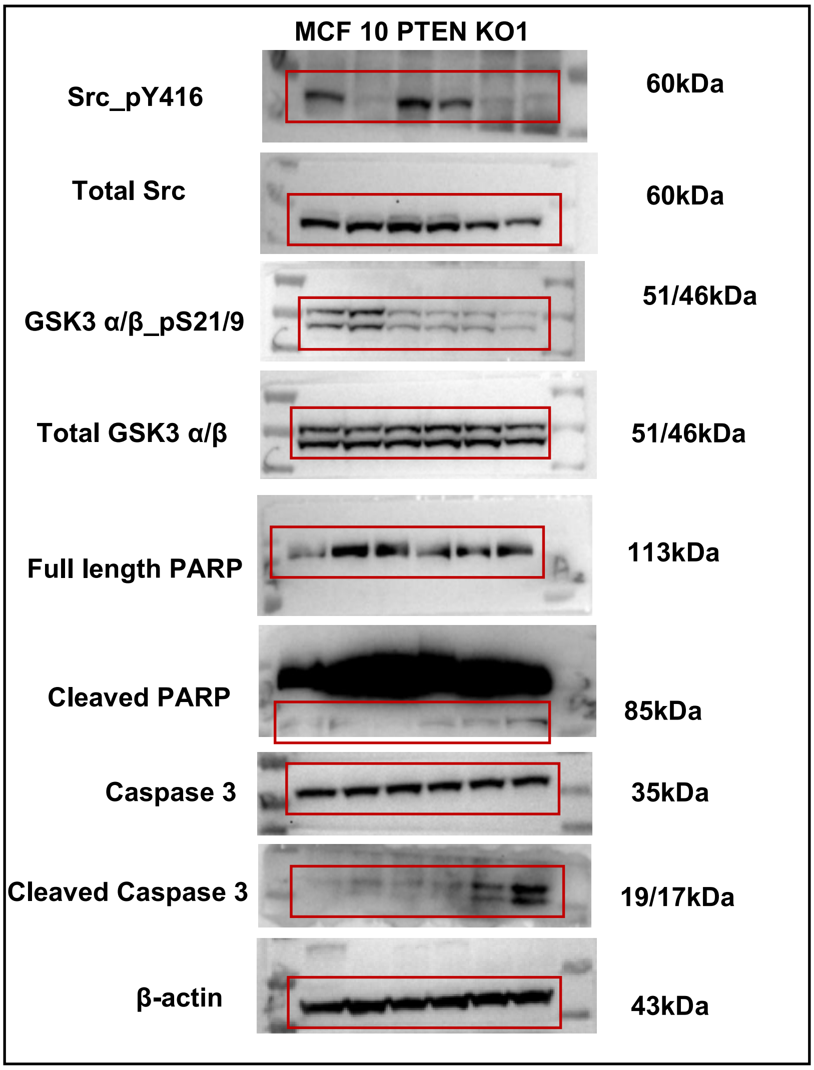
MCF 10 PTEN KO1
Src_pY416
60kDa
Total Src
60kDa
GSK3 α/β_pS21/9
51/46kDa
Total GSK3 α/β
51/46kDa
Full length PARP
113kDa
Cleaved PARP
85kDa
Caspase 3
35kDa
Cleaved Caspase 3
19/17kDa
β-actin
43kDa

Fig 8E

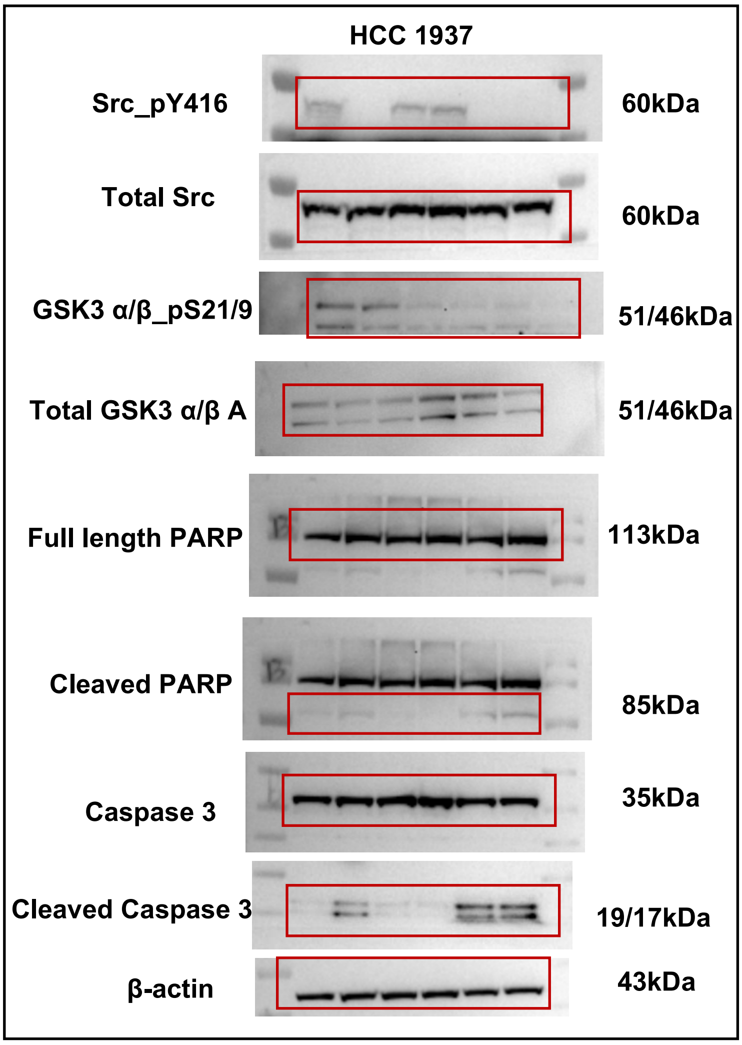
HCC 1937
Src_pY416
60kDa
Total Src
60kDa
GSK3 α/β_pS21/9
51/46kDa
Total GSK3 α/β A
51/46kDa
Full length PARP
113kDa
Cleaved PARP
85kDa
Caspase 3
35kDa
Cleaved Caspase 3
19/17kDa
β-actin
43kDa

Fig 8F

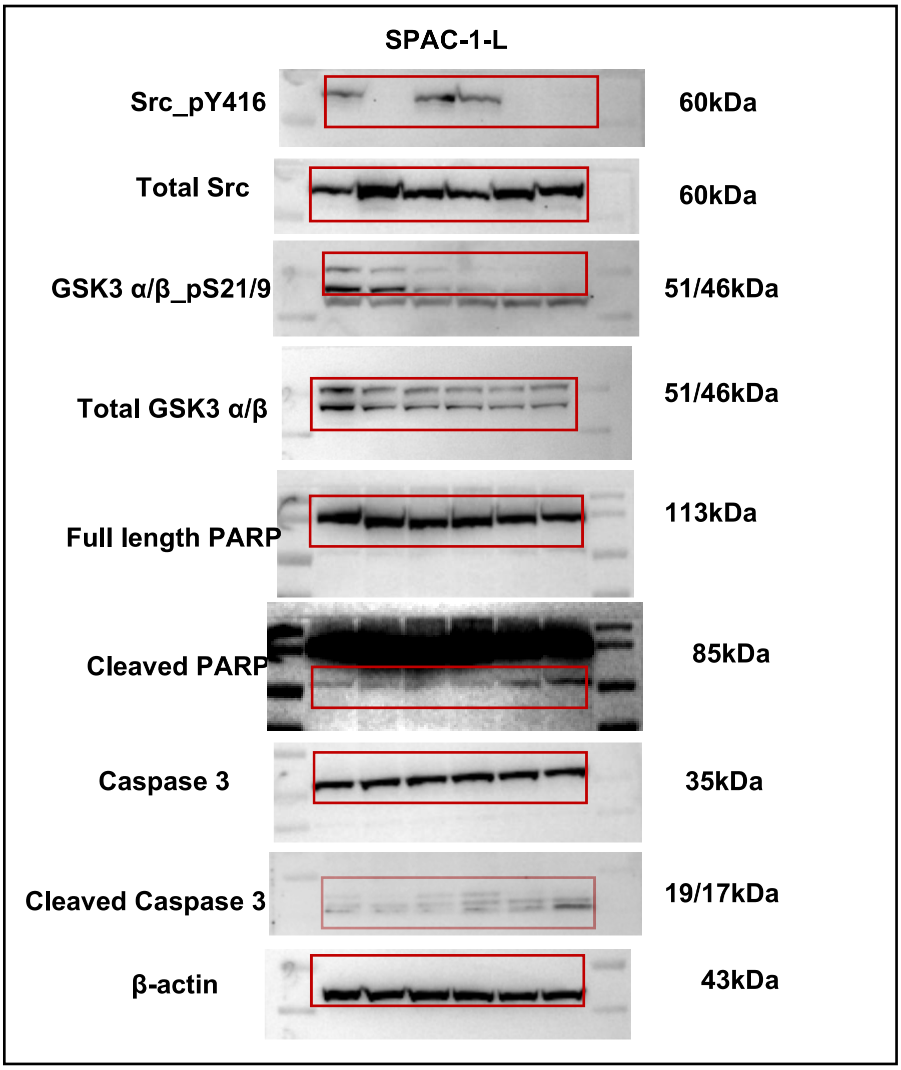
SPAC-1-L
Src_pY416
60kDa
Total Src
60kDa
GSK3 α/β_pS21/9
51/46kDa
Total GSK3 α/β
51/46kDa
Full length PARP
113kDa
Cleaved PARP
85kDa
Caspase 3
35kDa
Cleaved Caspase 3
19/17kDa
β-actin
43kDa

Fig. S1B

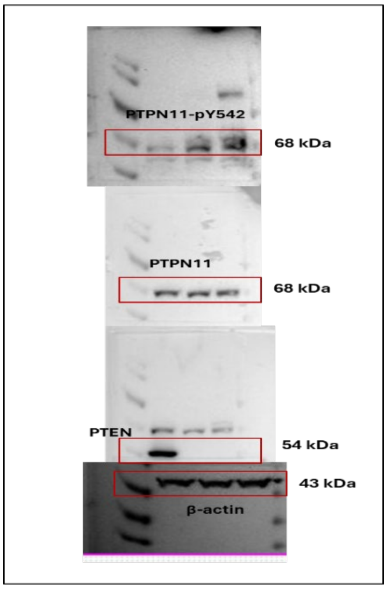

Fig. S5.B

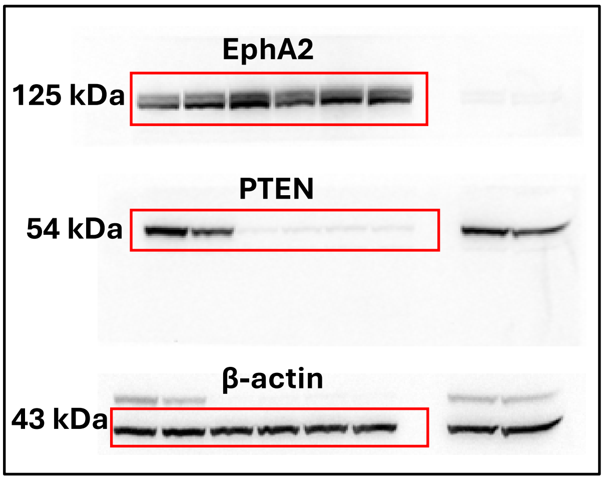

Fig. S6.A

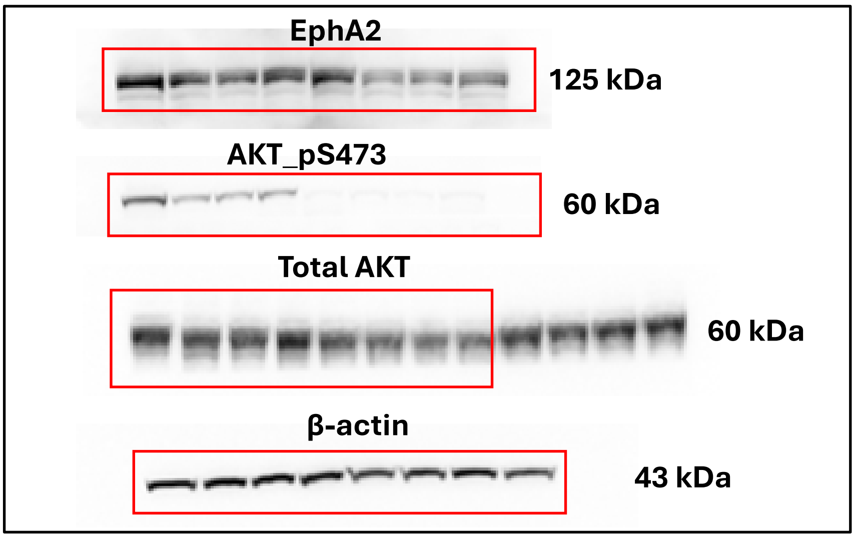

Fig. S6.B

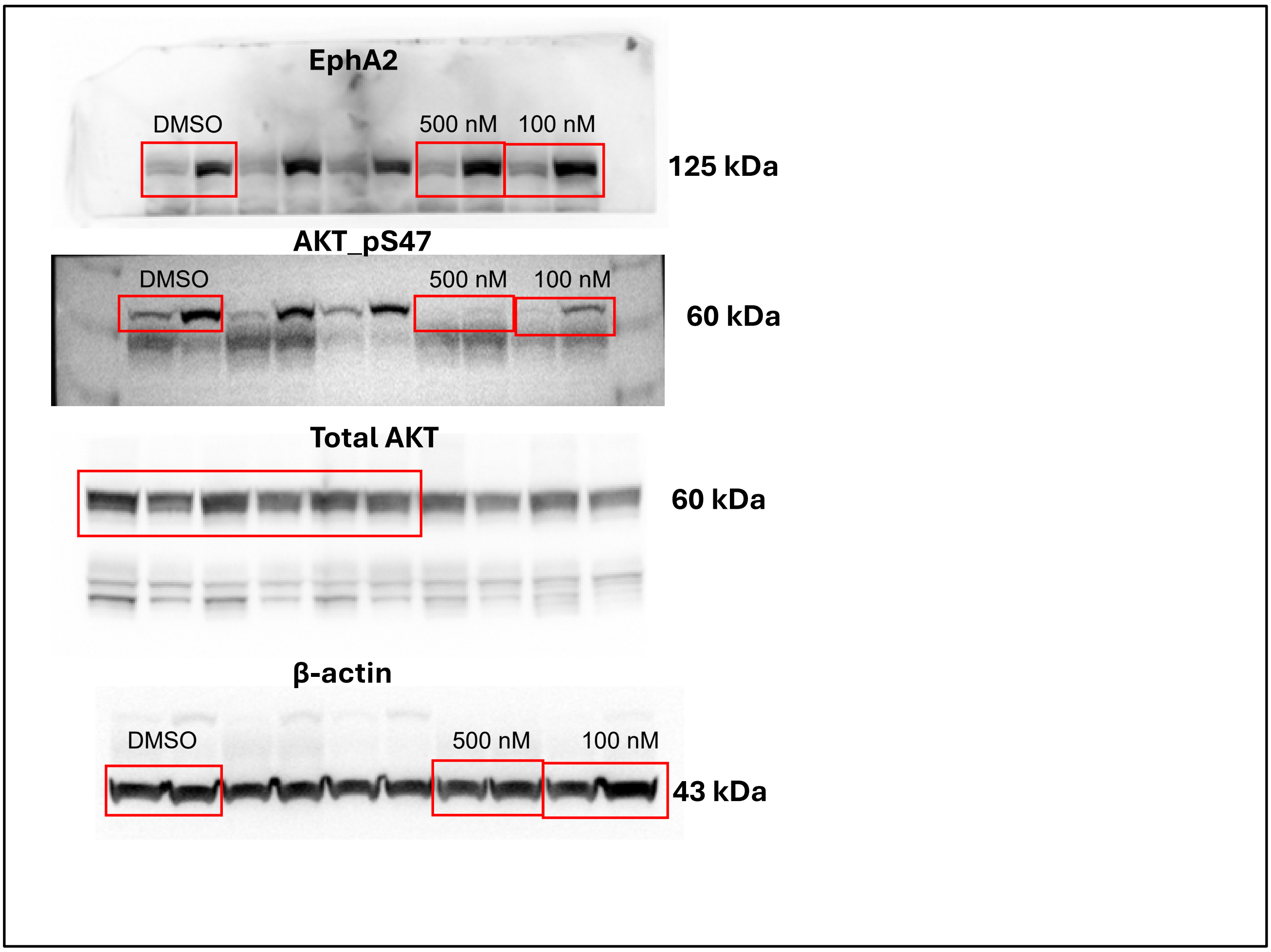
EphA2
DMSO
500 nM
100 nM
125 kDa
AKT_pS47
DMSO
500 nM
100 nM
60 kDa
Total AKT
60 kDa
β-actin
DMSO
500 nM
100 nM
43 kDa

Fig. S6.C

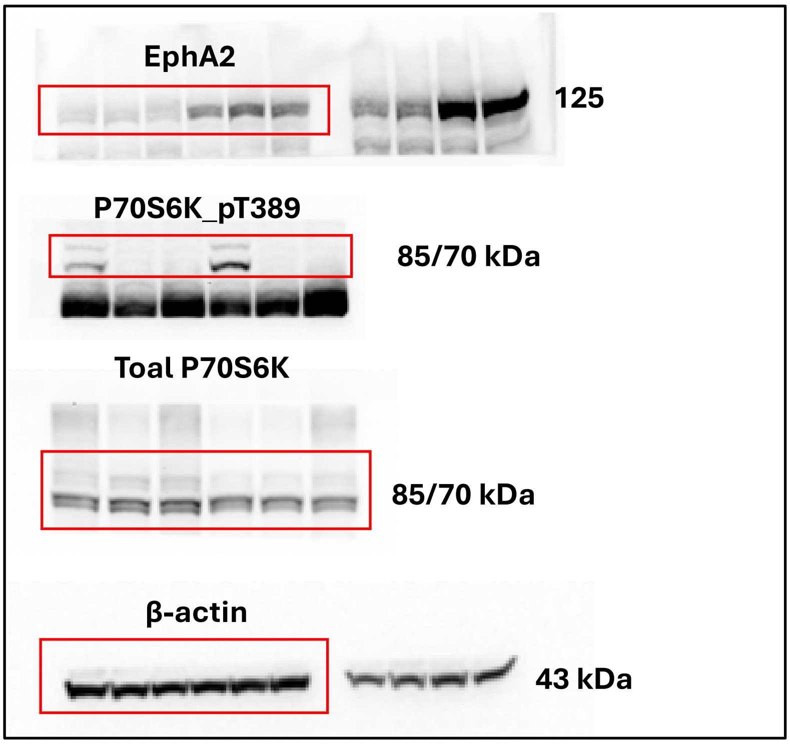

Fig. S6.D

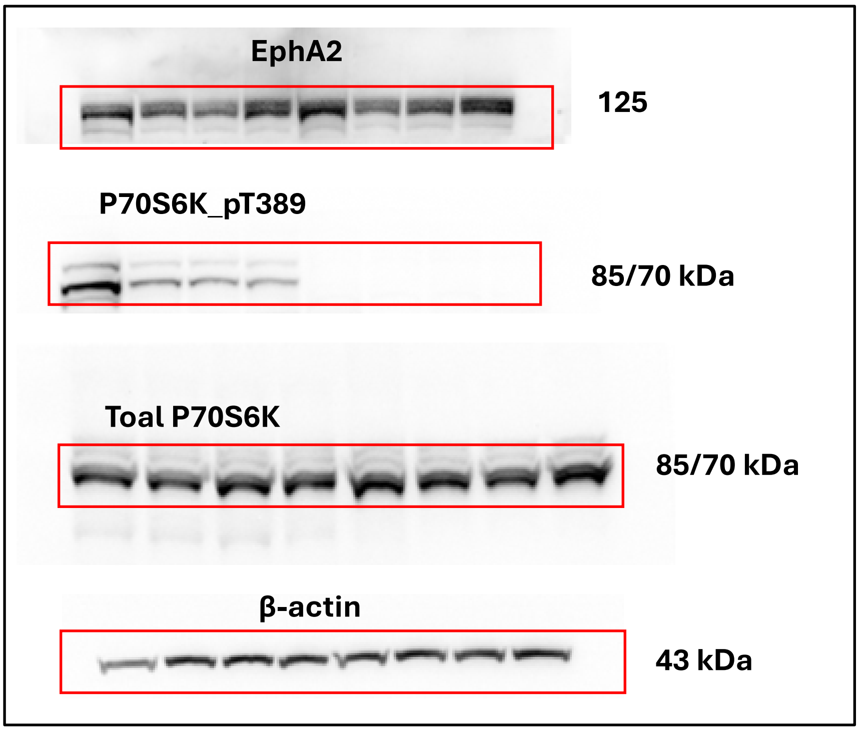

Fig. S6.E

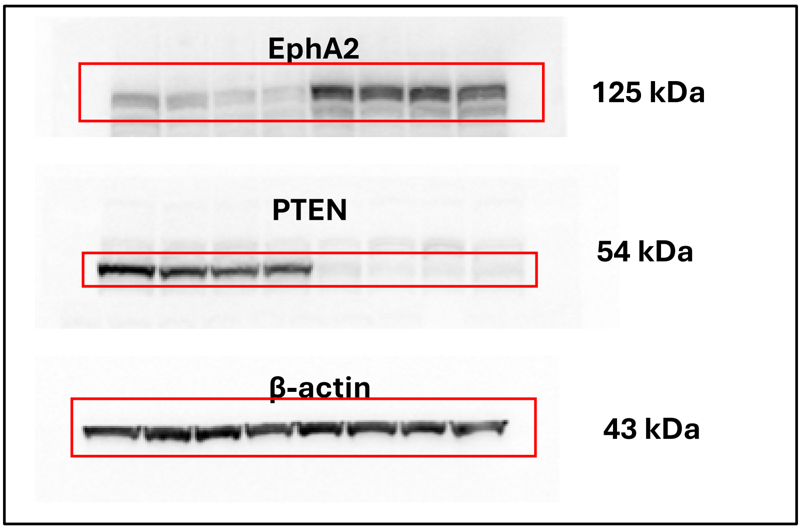

Fig. S6.F

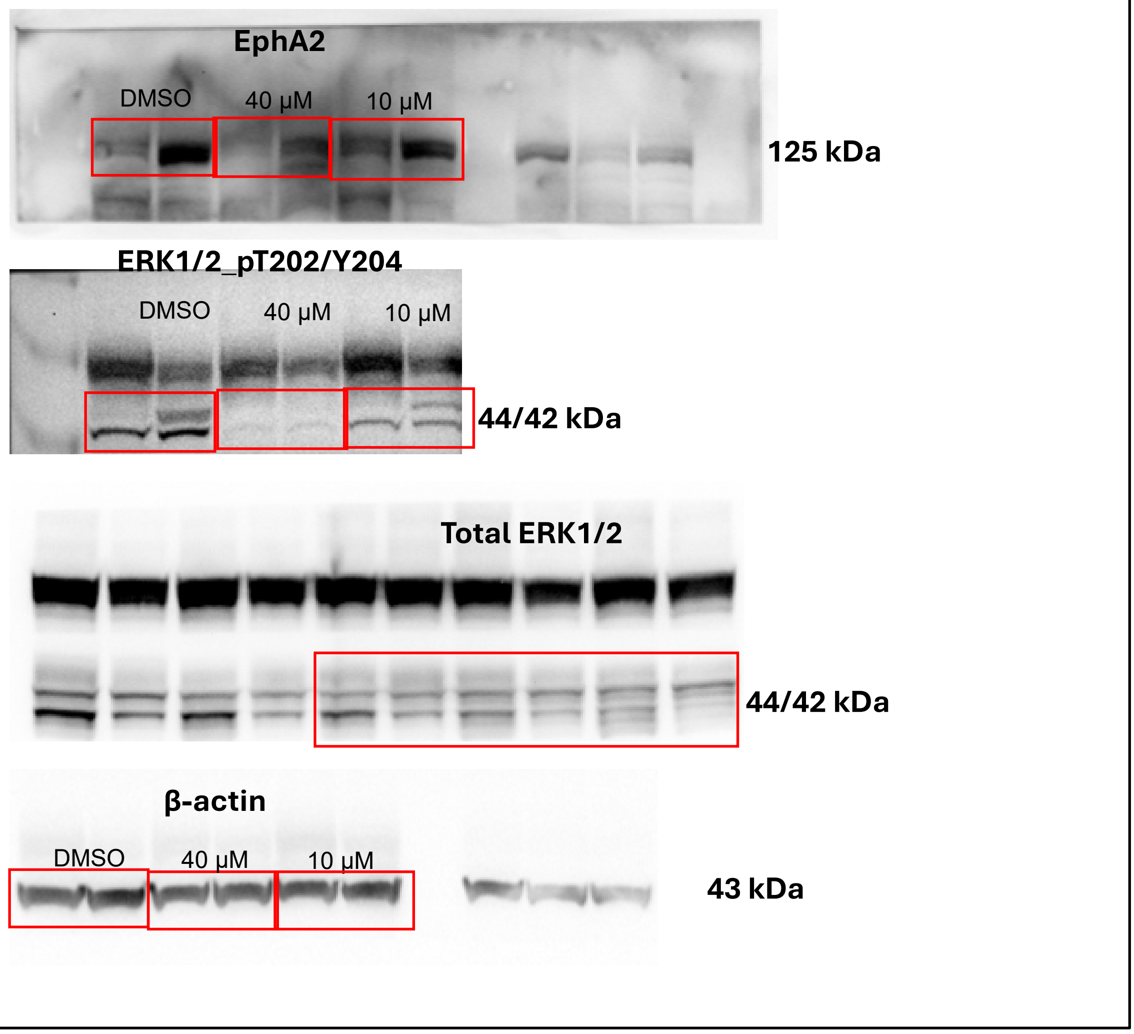

Fig. S6.G

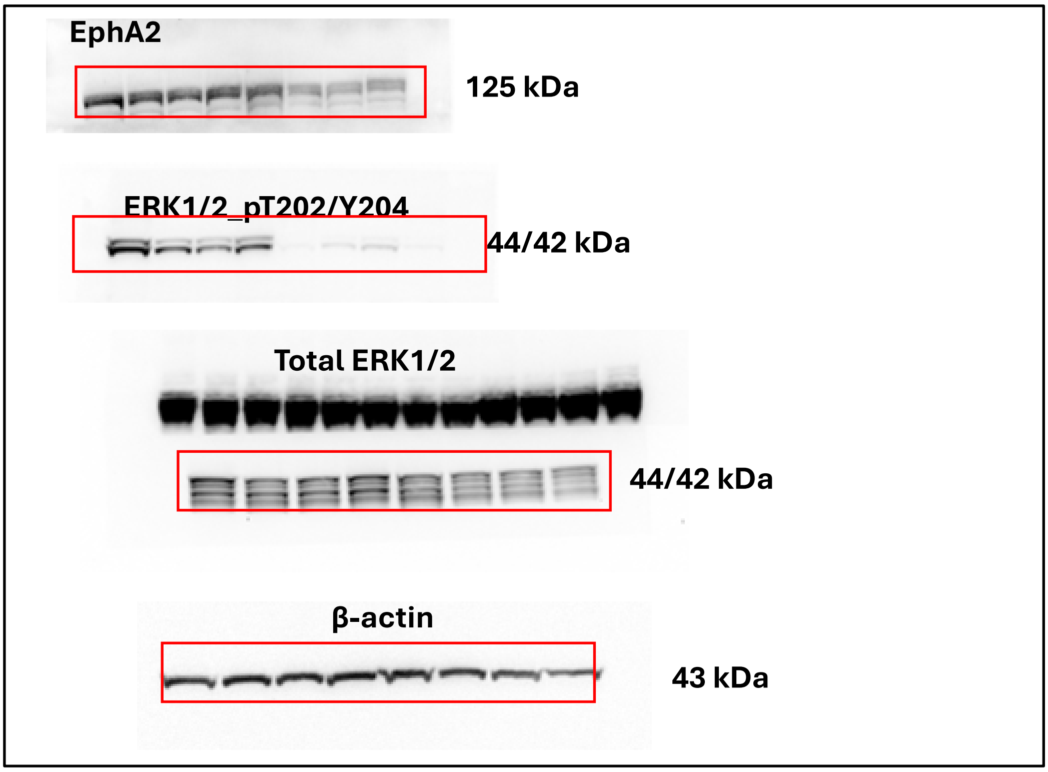
EphA2
125 kDa
ERK1/2_pT202/Y204
44/42 kDa
Total ERK1/2
44/42 kDa
β-actin
43 kDa

Fig. S7

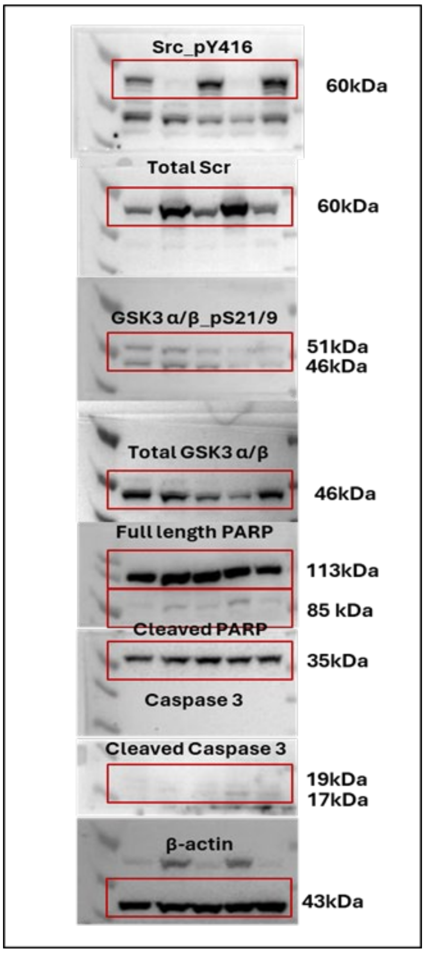

Src_pY416
60kDa
Total Scr
60kDa
GSK3 α/β_pS21/9
51kDa
46kDa
Total GSK3 α/β
46kDa
Full length PARP
113kDa
85 kDa
Cleaved PARP
35kDa
Caspase 3
Cleaved Caspase 3
19kDa
17kDa
β-actin
43kDa
