# Supplementary material for: Proteomic Analysis of PTEN-Deficient Cells Reveals Src-Mediated Upregulation of EphA2 and Therapeutic Potential of Dual Inhibition
Source: Mol Cell Proteomics. 2025 Oct 21;24(12):101316. doi: 10.1016/j.mcpro.2025.101316 (PMC12686823; doi:10.1016/j.mcpro.2025.101316)
Supplement: Supplementary Figures [file mmc11.pdf]

Proteomic Analysis of PTEN-Deficient Cells Reveals Src-Mediated Upregulation of EphA2 and Therapeutic Potential of Dual Inhibition

Authors:

Qiong Wang[1†], Xiangyi Kong[2,1†], Hongming Song[1,3†], Li Wang[1], Linrui Li[1], Xiaonan Hou[4], Santosh Renuse[5,13], Muhammad Saddiq Zahari[12,13], Ran Cheng[1], Md Kamrul Hasan Khan[1], Jidong Wang[1,6], Kiran Mangalaparthi[4], Lin Fang[7], Tamara Levin Lotan[8], Ben Ho Park[9], S. John Weroha[4], Huaijun Zhou[2*], Akhilesh Pandey[5,10,11*], Xinyan Wu[1,5*]

Affiliations:

[1]Department of Molecular Pharmacology and Experimental Therapeutics, Mayo Clinic, Rochester, MN 55905, USA

[2]Department of Obstetrics and Gynecology, Nanjing Drum Tower Hospital Clinical College of Nanjing Medical University, Nanjing, Jiangsu, 210008, China

[3]Breast Disease Center, The Affiliated Hospital of Qingdao University, Qingdao, Shangdong 266100, China

[4]Department of Oncology, Division of Medical Oncology, Mayo Clinic, Rochester, MN 55905, USA.

[5]Department of Laboratory Medicine and Pathology, Mayo Clinic, Rochester, MN 55905, USA

[6]Department of Obstetrics and Gynecology, Jinan Central Hospital Affiliated to Shandong University, Jinan, Shandong 250013, P.R. China.

[7]Department of Breast and Thyroid Surgery, Shanghai Tenth People's Hospital, Tongji University School of Medicine, Shanghai 200072, China.

[8]Department of Pathology, The Johns Hopkins Medical Institutions, Baltimore, MD. 21231, USA

[9]Division of Hematology, Oncology, Department of Medicine, Vanderbilt University Medical Center and the Vanderbilt-Ingram Cancer Center, Nashville, TN, USA.

[10]Center for Individualized Medicine, Mayo Clinic, Rochester, MN 55905, USA

[11]Manipal Academy of Higher Education (MAHE), Manipal, Karnataka, India

[12]Department of Biological Chemistry, Johns Hopkins Medical Institutions, Baltimore, MD. 21231, USA

[13]AstraZeneca, Rockville, MD. 20878, USA

† Q. Wang, X Kong, H Song contributed equally to this work.

* X Wu, A Pandey and H Zhou co-corresponding authors

Fig. S1

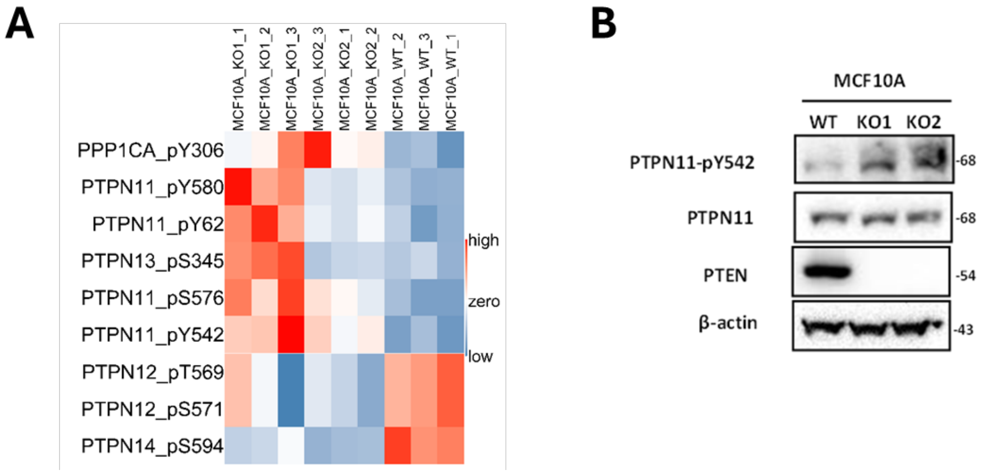

Fig. S1. **Phosphorylation of protein tyrosine phosphatases.** (**A**). A list of phosphorylated STY sites identified on phosphatases were significantly up- or downregulated by PTEN (Student's T-test p value < 0.05; fold change >= 1.5). (**B**). Western blot analysis was performed to examine the phosphorylation level of PTPN11 Y542 in MCF10A and PTEN KO clones.

Fig. S2

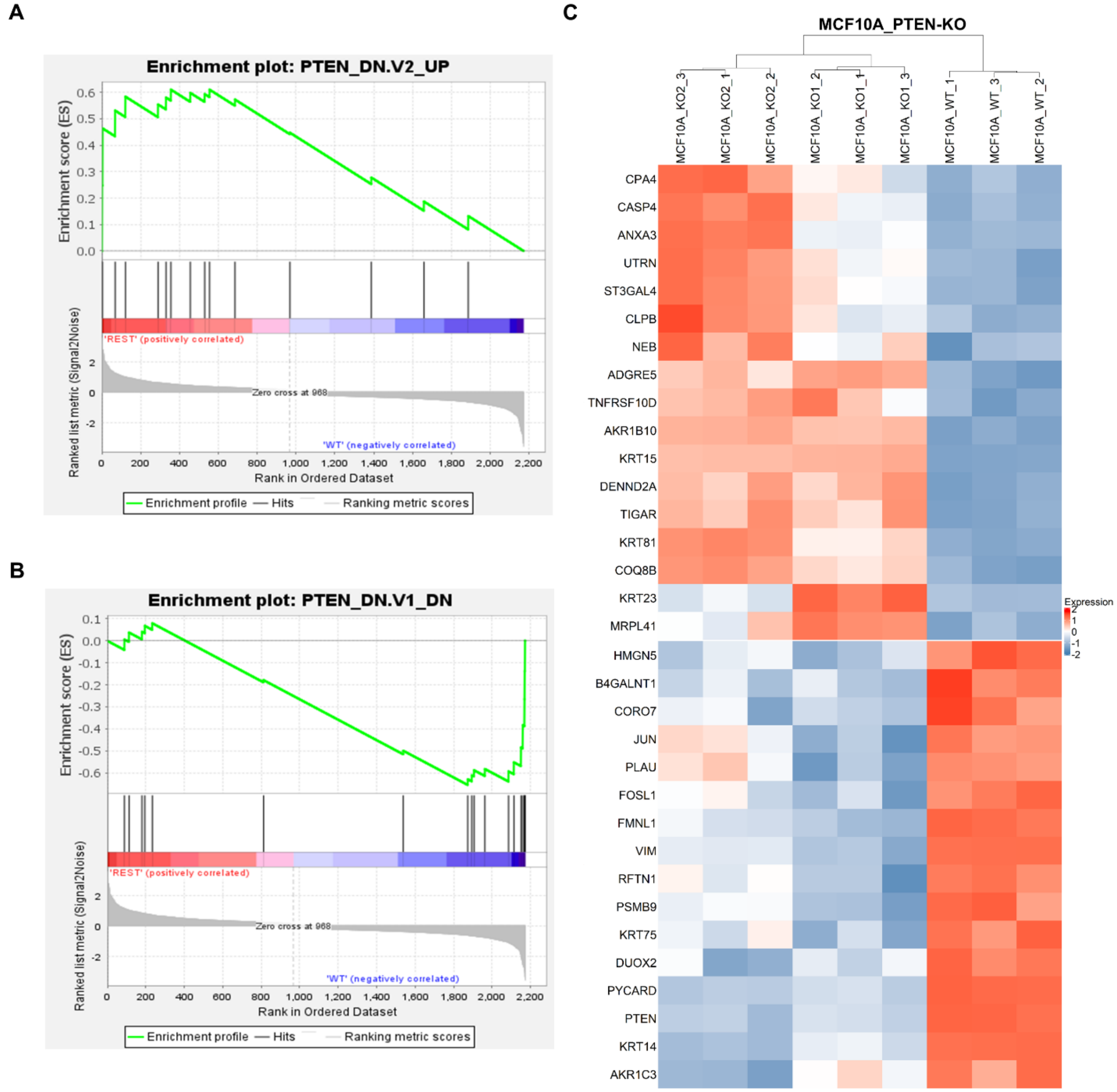

Fig. S2. **Enrichment plots of the enriched PTEN gene sets and their gene set members in GSEA.** The gene set members in the analyzed and enriched gene sets “PTEN_DN.V2_UP” **(A)** and “PTEN_DN.V1_DN” **(B)** that were included in the proteomic dataset were plotted in the heatmap **(C)**. Red: protein upregulation; Blue: protein downregulation.

Fig. S3

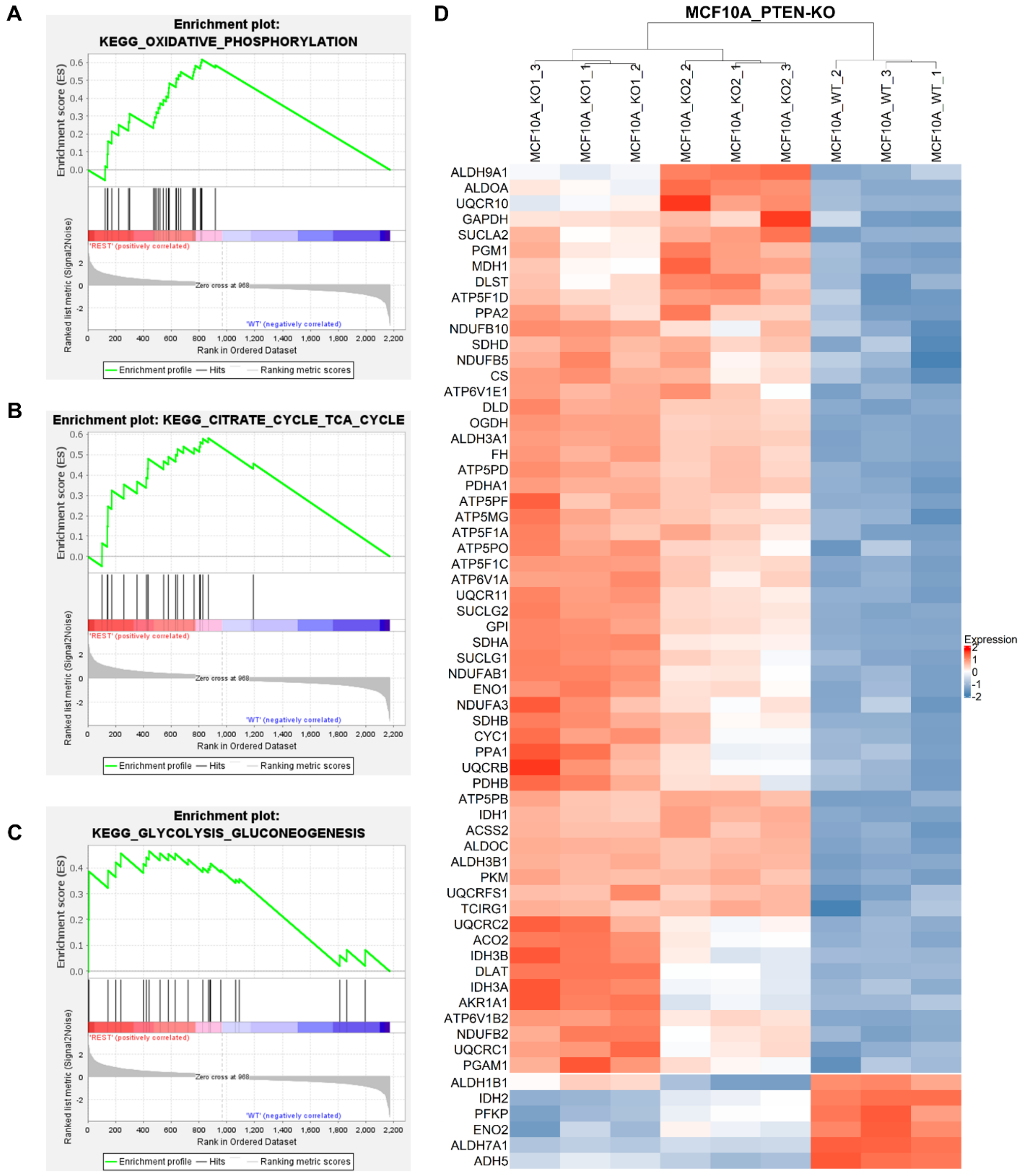

Fig. S3. **Enrichment plots of the enriched central carbon metabolic gene sets and their gene set members in GSEA.** The gene set members in the analyzed and enriched central carbon metabolism gene sets "KEGG_OXIDATIVE_PHOSPHORYLATION" **(A)**, "CITRATE_CYCLE_TCA_CYCLE" **(B)**, and "KEGG_CLYCOLYSIS_GLUCONEOGENESIS" **(C)** that were also included in the proteomic dataset were plotted in the heatmap **(D)**. Red: protein upregulation; Blue: protein downregulation.

Fig. S4

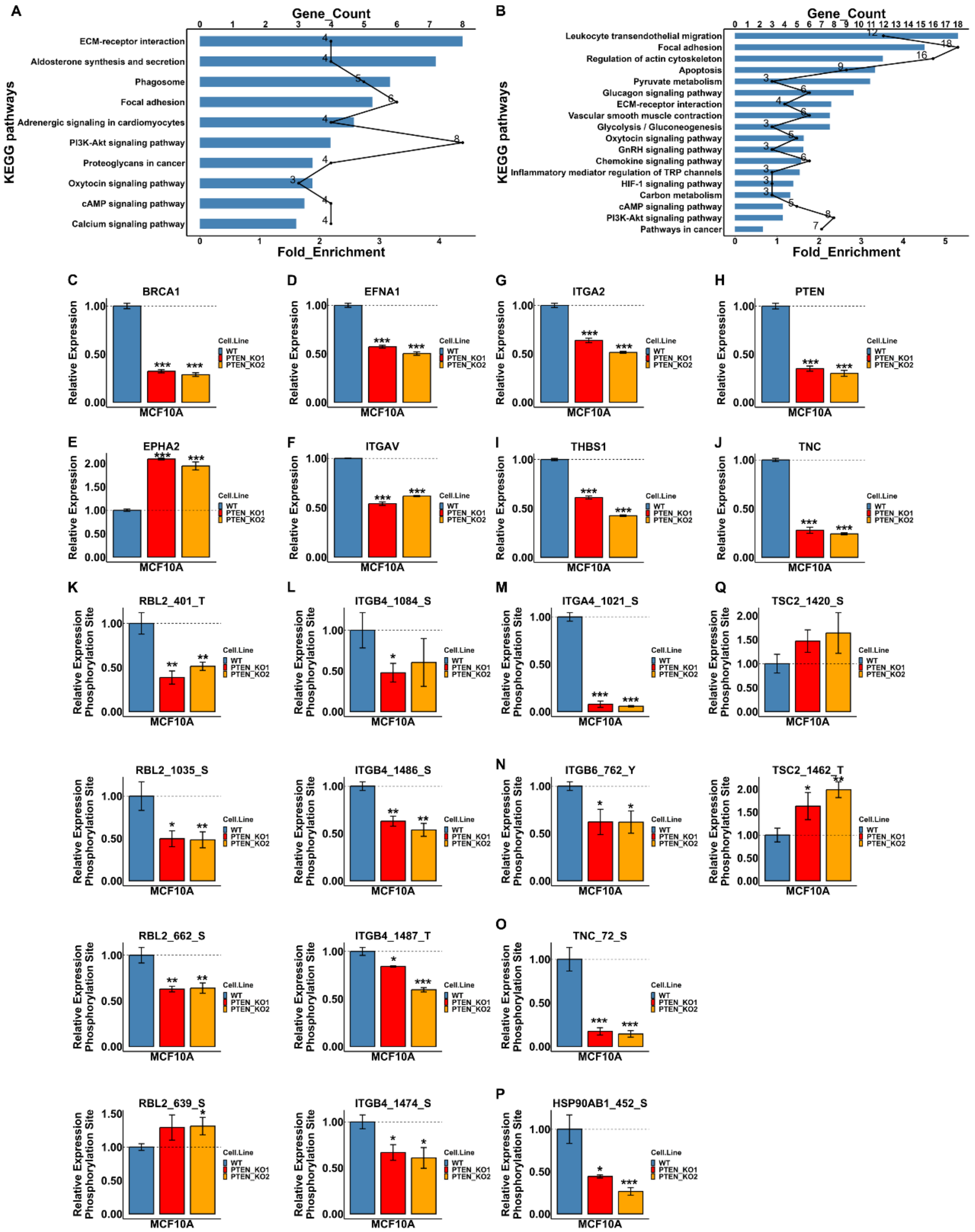

Fig. S4. **KEGG pathway enrichment and the identified significantly dysregulated PI3K-AKT signaling components**. **(A-B)** Significantly changed proteins **(A)** and phosphoproteins **(B)** (Student's T-test p value < 0.05; fold change >= 1.5) were analyzed for KEGG pathway enrichment in DAVID (Version: Dec 2021). Default parameter setup on DAVID was applied. Bar: fold enrichment; Line: gene count in each enriched pathway. **(C-Q)** Gene components in the PI3K-AKT KEGG signaling pathway were selectively plotted for their protein and/or phosphorylation levels detected in this study. Protein/phosphoprotein expression levels of *PTEN* knockout were normalized to the wide type. Two sample Student's T-test were performed for each knockout clone compared with the wide type. *: p value < 0.05; **: p value < 0.01; ***: p value < 0.001.

Fig. S5

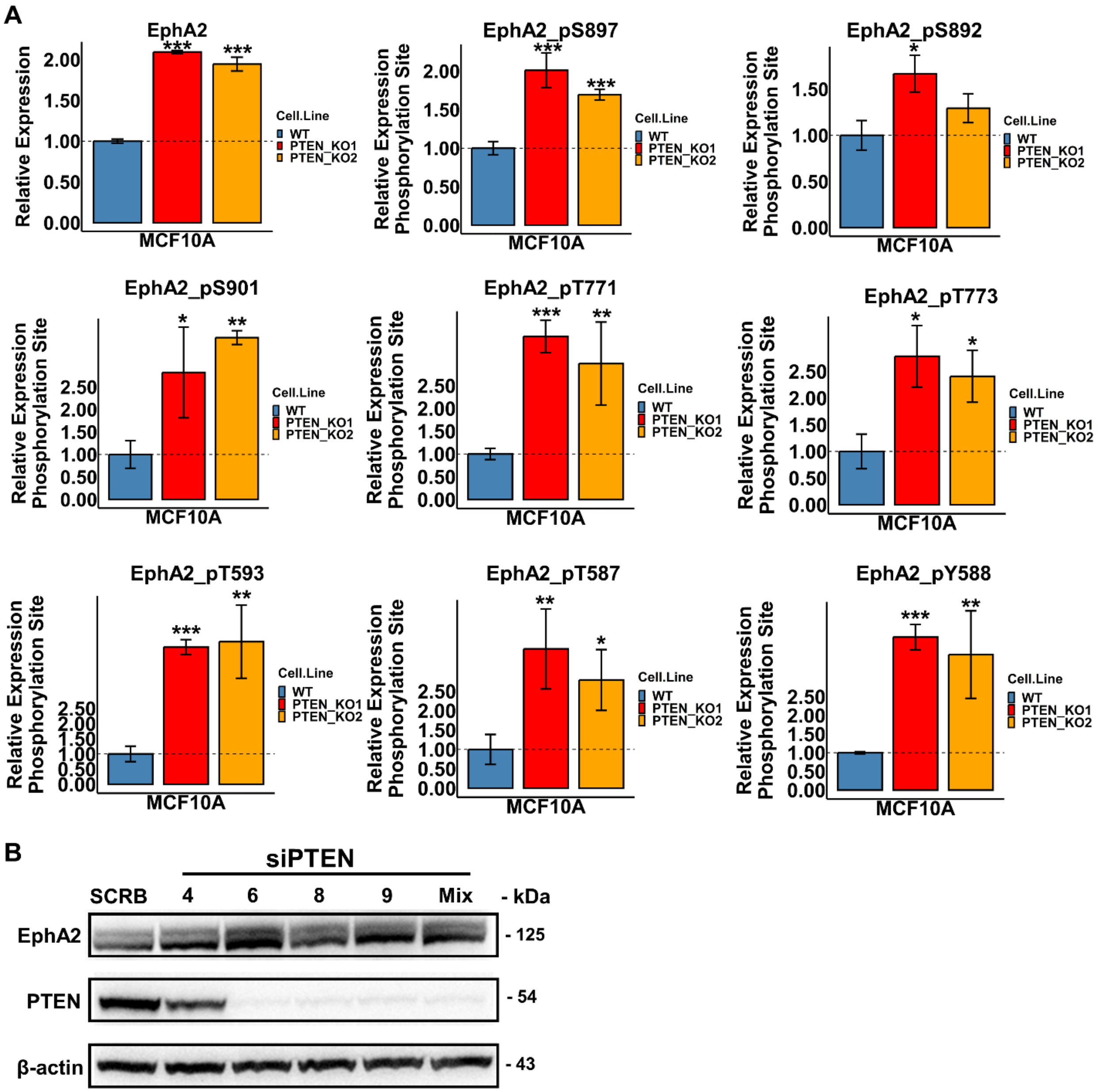

Fig. S5. **Functional assays for PTEN and EphA2. (A)** Total protein expression of EphA2 and levels of the phosphorylated sites detected on EphA2 by IMAC- and p-Tyr-1000 enrichment were plotted. **(B)** Knockdown efficiency of the siRNAs targeting PTEN.

Fig. S6

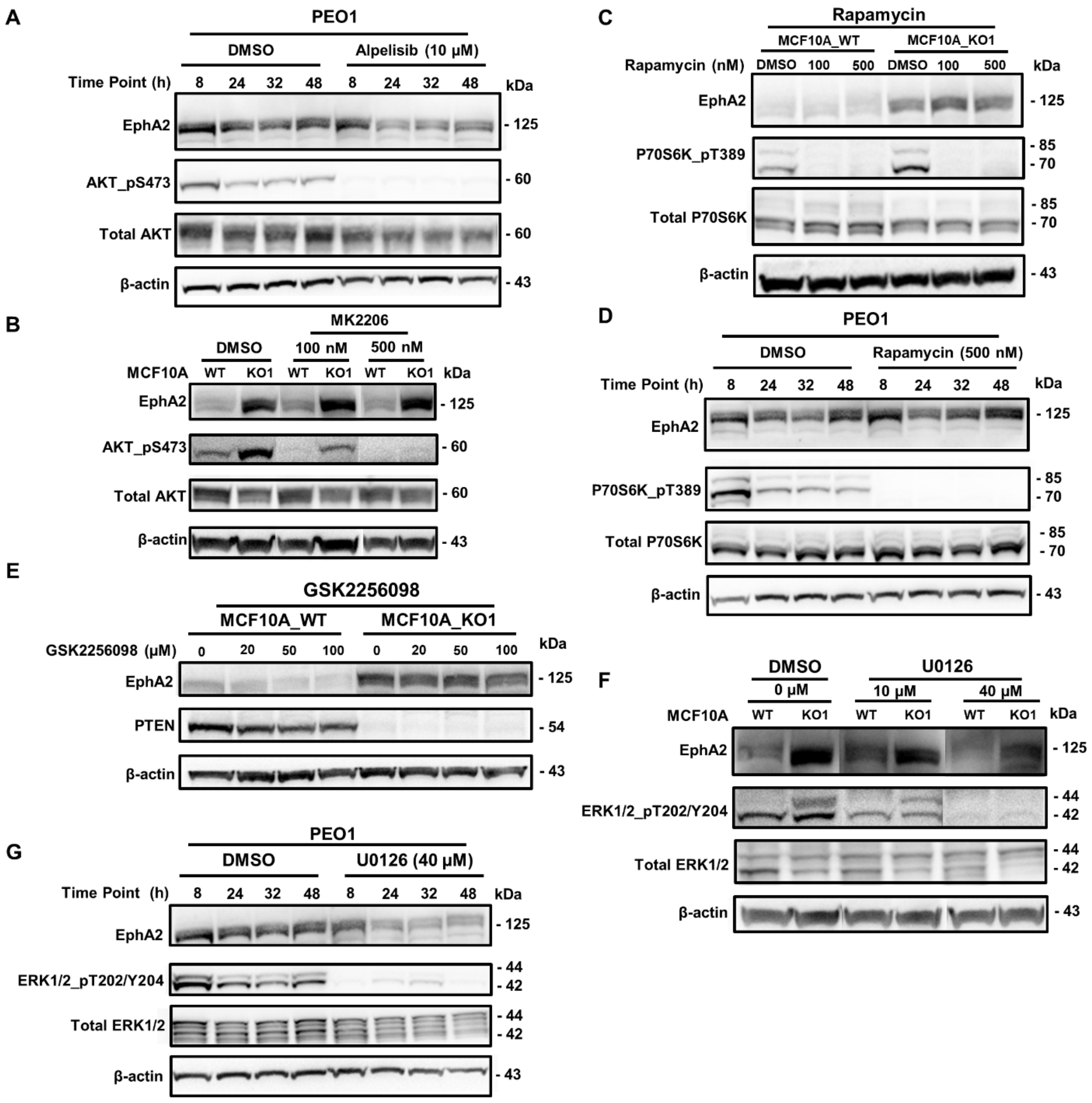

Fig. S6. **The mechanism of PTEN in regulating EphA2 protein expression. (A)** Time series of the PI3K inhibitor Alpelisib (10 μM) treatments in PEO1. **(B)** MCF10A wide type and PTEN knockout cell clone 1 were treated with the AKT inhibitor MK2206 (100 nM and 500 nM). **(C)** MCF10A wide type and PTEN knockout cell clone 1 were treated with the mTOR inhibitor Rapamycin (100 nM and 500 nM). **(D)** Time series of the mTOR inhibitor Rapamycin (500 nM) treatments in PEO1. **(E)** MCF10A wide type and PTEN knockout cell clone 1 were treated with the FAK1 inhibitor GSK2256098 (20 μM, 50 μM and 100 μM). **(F)** MCF10A wide type and PTEN knockout cell clone 1 were treated with the MEK inhibitor U0126 (10 μM and 40 μM). **(G)** Time series of the MEK inhibitor U0126 (10 μM and 40 μM) treatments in PEO1.

Fig. S7

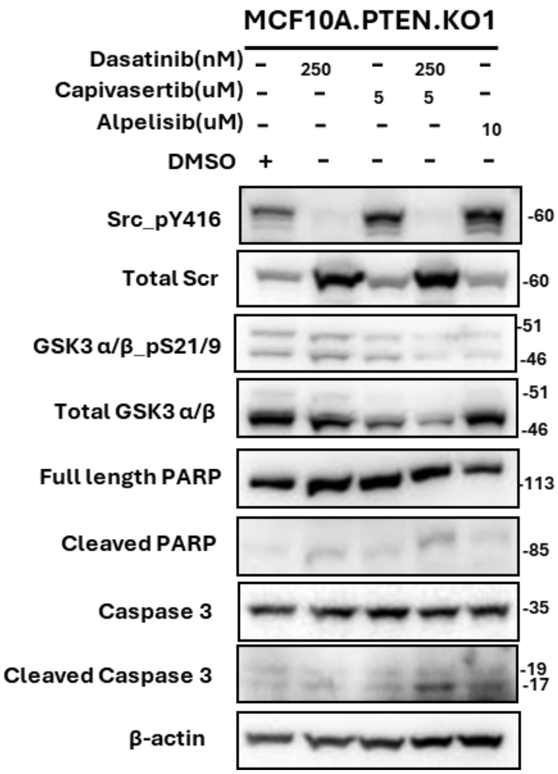

Fig S7. Alpelisib does not suppress Src Y416 phosphorylation. MCF10A-PTEN-KO1 cells were treated with DMSO, dasatinib, capivasertib or alpelisib, respectively. Western blots were performed to examine the indicated protein phosphorylations and total expressions.

Fig. S8

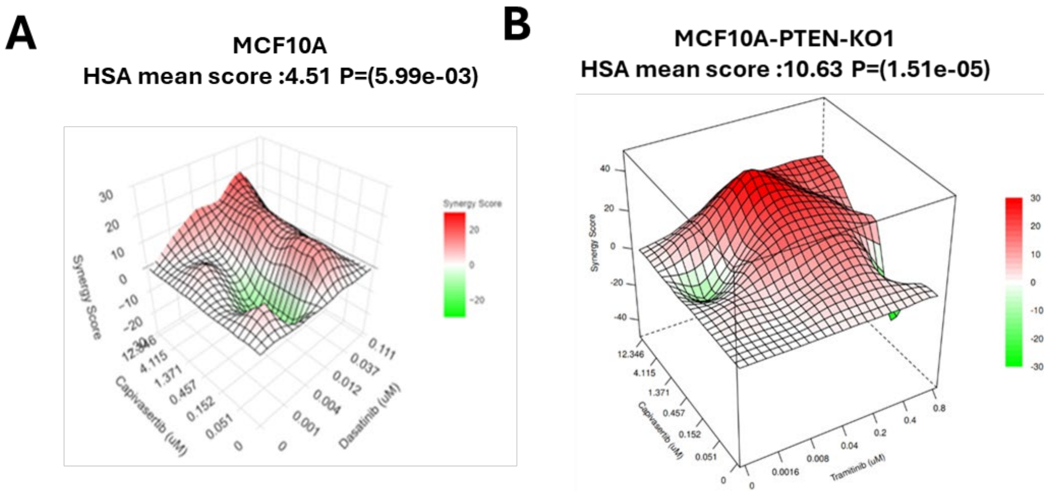

Fig. S8. **Synergy Analysis**. (**A**). MCF 10A cells were combinatorially treated with different concentrations of dasatinib and capivasertib. (**B**). MCF10A-PTEN-KO1 cells were combinatorially treated with different concentrations of trametinib and capivasertib. High synergy scores (HSA, red regions) indicate synergistic effects and HSA mean score > 10 indicating synergistic interactions.

Fig. S9

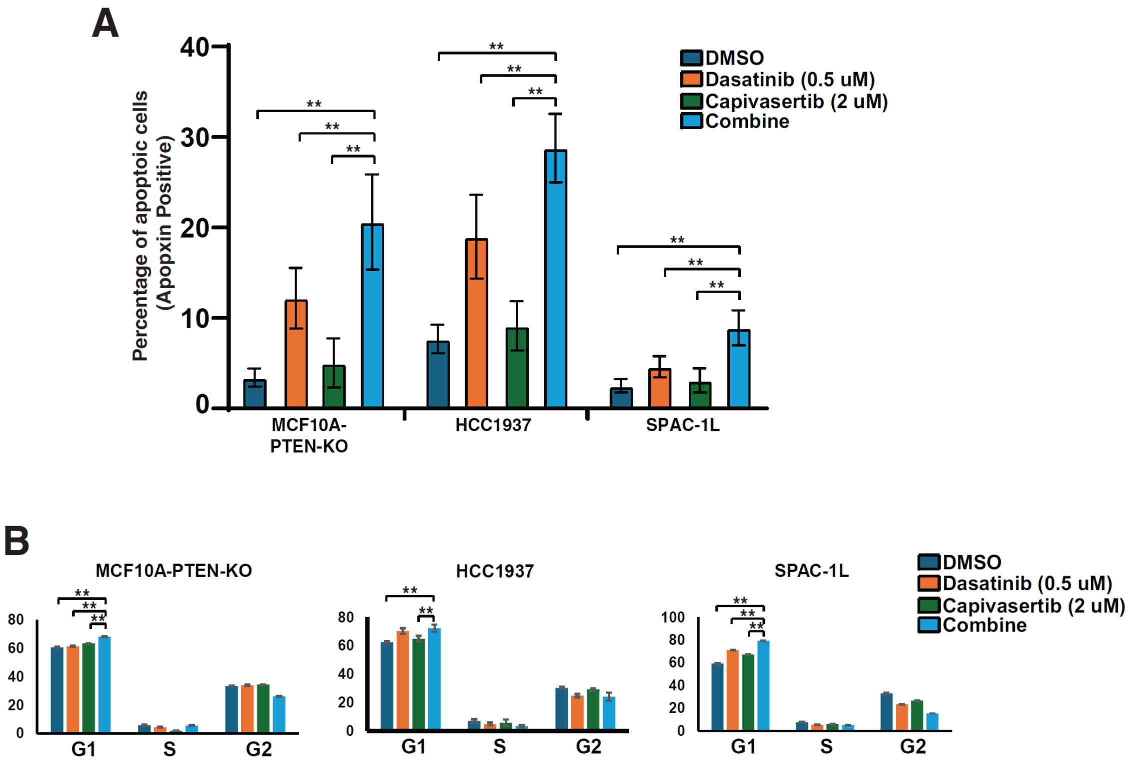

Fig. S9. **Inhibiting SRC and AKT kinase activity significantly induce apoptosis and cell cycle arrest for PTEN deficient cells. (A)** Apoptosis analysis of MCF10A-PTEN-KO1, HCC1937, and SPAC-1-L cells treated with DMSO, dasatinib (0.5 μM), capivasertib (2 μM), or the combination. Apoptotic cells were quantified by Apopxin staining. Percentage of Apopxin positive cells were calculated. Combination treatment significantly increased apoptosis compared with single agents. **(B)** Cell cycle distribution of the same cells following treatment with DMSO, dasatinib, capivasertib, or the combination. Co-inhibition of SRC and AKT induced marked G1 arrest in PTEN-deficient cells. Data are presented as mean ± SD. Statistical significance was determined using Student's t-test (**p < 0.01).

Supplemental Data

Supplemental Data 1. A complete list of proteins identified in MCF10A cells with PTEN KO.

The output data table "Proteins" in the result report from Proteome Discoverer was shown. It listed all the identified master proteins in the MCF10A, MCF10A_PTEN_KO1, and MCF10A_PTEN_KO2 samples. For each identified protein, the protein accession, protein name, gene name, protein coverage, information on peptides and spectra, protein quantification before and after normalization, protein modification, and the statistics of protein identification were reported.

Supplemental Data 2. A complete list of phosphorylated sites identified in MCF10A cells with PTEN KO.

Quantification results of the MCF10A, MCF10A_PTEN_KO1, and MCF10A_PTEN_KO2 samples in triplicates. Data were consolidated on the phosphorylation site level. Protein accession number, protein name, gene name, peptide and phosphorylation site information were provided along with the site quantification.

Supplemental Data 3: Quantitative information of proteins and phosphorylation sites identified in MCF10A wild type and PTEN KO clones.

The intensity of the complete list of identifiable and quantifiable proteins **(sheet 1)** and phosphorylaion sites **(sheet 2)** for all samples was imputed and normalized, followed by statistical analysis. Fold changes (PTEN wide type / knockout), p value, and FDR were reported. Consistency between the two knockout cell lines was also reported. Significant changes (FC >= 1.5 and p value < 0.05) were reported separately for total proteome expression **(sheet 3)** and phosphorylation **(sheet 4)**.

Supplemental Data 4: The identified protein kinases and phosphatases in the proteomic and phosphoproteomic datasets.

Kinases **(sheet 1-4)** and phosphatases **(sheet 5-6)** that were identified and quantified in the proteomic- **(sheet 1)** and phosphoproteomic **(sheet 2)** datasets were listed, along with the fold changes between PTEN wide type and knockout and their statistical information. The protein accession number, protein name, gene name, and the associated kinase group/family were provided. The kinase and phosphatase libraries used in this study were referenced from literature (Manning G, 2002, Science; Chen J., 2017, Science Signaling). Significant changes (p < 0.05) were reported separately for total proteome expression **(sheet 3)** and phosphorylation **(sheet 4)**. TP: total proteome; pST: phosphorylated serine and threonine.

Supplemental Data 5: KEGG pathway enrichment and KEA enrichment analysis.

KEGG pathway enrichment analysis was conducted for altered protein kinases **(sheet 1)**, altered general proteins **(sheet 4)**, and proteins with altered phosphorylation **(sheet 5)**. The ranked kinases that were enriched by KEA3 were listed based on two ranking methods (mean rank and top rank) **(sheet 2 and 3)**.

Supplemental Data 6: A list of phosphopeptides identified in the phosphotyrosine peptide enrichment-based proteomics analysis (SILAC).

The output data table "Peptide groups" in the result report from Proteome Discoverer was shown for the SILAC experiment. It listed all the identified phosphorylated peptides in the MCF10A, MCF10A_PTEN_KO1, and MCF10A_PTEN_KO2 samples. For each identified phosphopeptide, the related protein information, information on associated phosphorylation sites and spectra, statistics for identification, and peptide quantification were reported.

Supplemental Data 7: Quantitative and statistical information, along with biological analysis, of phosphorylated sites identified in MCF10A wild type and PTEN KO clones in the phospho-tyrosine enriched SILAC experiment.

The list of phosphorylation sites that were significantly changed ($p < 0.05$) were listed, along with fold changes (PTEN wide type / knockout) **(sheet 1)**. Kinases that were identified and quantified were also listed ($p < 0.05$, $|\text{fold change}| >= 1.5$) **(sheet 4)**. The ranked kinases that were enriched were listed by KEA3 based on two ranking methods (mean rank and top rank) **(sheet 2 and 3)**. SRC interacting proteins **(sheet 6)** and the altered phosphosites **(sheet 5)** from proteins identified as SRC interacting proteins were also reported.

Supplemental Data 8: Lists of protein kinases and phosphosites of protein kinases that were significantly altered in PTEN KO cells.

This table contains information of the main figure: the human kinase phylogenetic tree.

Protein kinases and phosphosites of protein kinases that were significantly altered in PTEN KO cells were reported based on data of total proteome **(sheet 1)**, IMAC-enriched phosphoproteome **(sheet 2)**, and phospho-tyrosine enriched phosphoproteome **(sheet 3)**. The protein accession number, protein name, gene name, and the associated kinase group/family were provided, along with the fold changes between PTEN wide type and knockout and their statistical information.

Supplemental Data 9: Clinical characteristics of the 3 endometrial cancer tumors

Staging was based on FIGO 2009 since all tumors were collected prior to the 2023 update. All carcinosarcomas are considered high grades and a numerical value for grade is not reported. Systemic or radiation therapy for endometrial cancer, if any, is only reported if it was administered prior to tumor collection.
